# FAK signalling controls insulin sensitivity through regulation of adipocyte survival

**DOI:** 10.1038/ncomms14360

**Published:** 2017-02-06

**Authors:** Cynthia T. Luk, Sally Yu Shi, Erica P. Cai, Tharini Sivasubramaniyam, Mansa Krishnamurthy, Jara J. Brunt, Stephanie A. Schroer, Daniel A. Winer, Minna Woo

**Affiliations:** 1 Toronto General Hospital Research Institute, University Health Network, MaRS Ctre, TMDT, 101 College Street, 10th Floor, Room 10-363, Toronto, Ontario, Canada M5G 1L7; 2 Institute of Medical Science, University of Toronto, Toronto, Ontario, Canada M5S 1A8; 3 Department of Pathology, University Health Network, Toronto, Ontario, Canada M5G 2C4; 4 Division of Endocrinology, Department of Medicine, University Health Network, University of Toronto, Toronto, Ontario, Canada M5G 2C4

## Abstract

Focal adhesion kinase (FAK) plays a central role in integrin signalling, which regulates growth and survival of tumours. Here we show that FAK protein levels are increased in adipose tissue of insulin-resistant obese mice and humans. Disruption of adipocyte *FAK* in mice or in 3T3 L1 cells decreases adipocyte survival. Adipocyte-specific *FAK* knockout mice display impaired adipose tissue expansion and insulin resistance on prolonged metabolic stress from a high-fat diet or when crossed on an obese *db/db* or *ob/ob* genetic background. Treatment of these mice with a PPARγ agonist does not restore adiposity or improve insulin sensitivity. In contrast, inhibition of apoptosis, either genetically or pharmacologically, attenuates adipocyte death, restores normal adiposity and improves insulin sensitivity. Together, these results demonstrate that FAK is required for adipocyte survival and maintenance of insulin sensitivity, particularly in the context of adipose tissue expansion as a result of caloric excess.

## Abstract (web summary)

The kinase FAK is important for integrin signalling and promotes cell survival. Here, the authors demonstrate FAK regulates adipocyte survival, and is particularly important for maintaining insulin sensitivity during adipose tissue expansion in the context of a calorie-rich diet.

Obesity is characterized by massive expansion of adipose tissue. Although obesity is the strongest risk factor for type 2 diabetes and insulin resistance, not all obese individuals are insulin resistant and many with diabetes are not obese, illustrating the complexity of adipose tissue biology and its relationship with metabolic dysfunction.

In an environment of caloric excess, expansion of adipose tissue can result in hypoxia, contributing to adipocyte cell death, which triggers chronic, low-grade inflammation, fibrotic extracellular matrix (ECM) accumulation and ultimately insulin resistance^1^,^2^,^3^. However, the precise nature of the links between these processes are unclear, with studies showing that cell death, inflammation and insulin resistance commonly cluster together but can also be disassociated under certain conditions^4^,^5^,^6^; thus, the intracellular mechanisms regulating the adipocyte response to tissue expansion and contributing to metabolic defects remain unclear.

Focal adhesion kinase (FAK) is a 125 kDA non-receptor tyrosine kinase essential for development and whole-body deletion of the FAK gene (*Ptk2*) is lethal at embyronic day 8.5 (ref. 7). FAK is best recognized for its central role in integrin signalling, which transmits signals from the ECM intracellularly. FAK is overexpressed in many human tumours and can play important roles in a variety of cell processes such as survival, invasion, migration and growth in this setting^8^,^9^,^10^. Major interactions for FAK include tumour suppressor p53 and extracellular signal-regulated kinase 1 (ERK1) and ERK2, both critical for conferring resistance to apoptosis^10^,^11^,^12^. However, little is known about the role of FAK in metabolic disease and tissue growth in other contexts such as energy excess, wherein adipose tissue is the most dynamic organ.

We find that in both mice and humans, FAK increases in adipocytes with obesity and insulin resistance, suggesting an important role of adipocyte FAK in metabolism. Disrupting *FAK* specifically in adipose tissue of mice results in insulin resistance, with increased adipocyte cell death and impaired adipose tissue expansion with high-fat diet (HFD) feeding, as well as in genetically obese *db/db* and *ob/ob* models. Inhibiting apoptosis in mice with adipocyte FAK deficiency, either via concomitant *Casp3*^*+/−*^, *Casp3*^*−/−*^ or adipose tissue-specific *Casp8*^*−/−*^ mutations, or treatment with apoptosis inhibitor, restored insulin sensitivity and adiposity, demonstrating an essential role of FAK signalling in adipocyte survival that is required for appropriate adipocyte expansion and maintenance of insulin sensitivity during metabolic stress.

## Results

### Adipocyte FAK increases with obesity and insulin resistance

**Figure 1:**
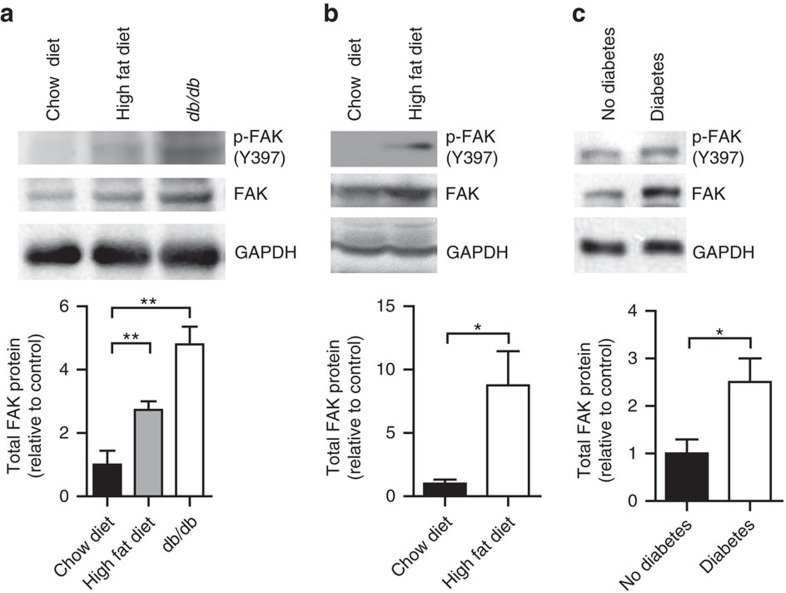
FAK increases in adipose tissue with obesity and insulin resistance. (**a**,**b**) Representative blot and quantification of FAK protein in adipocytes isolated from perigonadal WAT (*n*=4 mice) (**a**) and interscapular BAT (*n*=5 mice) (**b**) of 20-week-old mice fed HFD or *db/db* mice relative to chow diet-fed mice. (**c**) Representative blot and quantification of FAK in protein lysates from omental adipocytes of humans with and without type 2 diabetes (DM) (*n*=3 humans). Data are mean±s.e.m. *P<0.05 and **P<0.01 by Student's *t*-test.

In an environment of energy excess, adipose tissue is uniquely capable of undergoing massive growth and expansion. Many studies have shown that FAK plays an important role in tumour growth^9^,^10^, but have not explored its role in tissue expansion outside this context. To determine whether FAK could play a role in the setting of obesity and insulin resistance, we examined FAK protein levels in both mice and humans. FAK protein and gene expression increased about threefold in adipocytes isolated from perigonadal white adipose tissue (WAT) from mice fed a HFD for 12 weeks and about fourfold in genetically obese diabetic *db/db* mice compared with adipocytes from chow diet-fed wild-type control littermates (Fig. 1a and Supplementary Fig. 1a). FAK protein also increased 8.8-fold in interscapular brown adipose tissue (BAT) from mice fed HFD (Fig. 1b). This increase in FAK was not seen in stromal vascular cells containing macrophages and preadipocytes (Supplementary Fig. 1b). Similarly, omental adipocytes from humans with type 2 diabetes showed 2.5-fold increased expression of FAK when compared with humans without diabetes (Fig. 1c). Phosphorylation of FAK at major site of activation, Tyr397, also appeared increased, but this was in proportion to the increase in total FAK (Fig. 1a–c and Supplementary Fig. 1c–e). Overall, this identifies that in both common mouse models of obesity and diabetes, and in humans with metabolic dysfunction, FAK expression is upregulated in adipocytes.

### FAK is required for adipocyte survival

Particularly in cancer cells, FAK has been implicated in playing an important role in many processes, such as migration, invasion, growth and survival^10^. To definitively study the role of FAK in adipose tissue *in vivo*, we used aP2 or adiponectin Cre-loxP systems^13^,^14^,^15^ to generate novel mouse models with adipocyte-specific knockdown of *FAK* (*aP2FAK*^*−/−*^ or *adipoqFAK*^*−/−*^ mice, respectively). *aP2FAK*^*−/−*^ mice had significantly decreased *FAK* gene and protein expression in isolated adipocytes and fat pads compared with control mice (Supplementary Fig. 2a–c). Knockdown was seen in all adipose tissue fat pads, including inguinal WAT, perigonadal WAT and interscapular BAT (Supplementary Fig. 2c). Owing to potential concerns about aP2 promoter activity in other tissues including macrophages or during development, we also measured FAK levels in multiple other tissues and in macrophages and did not see significant deletion of FAK (Supplementary Fig. 2d,e), consistent with recent studies using this Cre-loxP strain^13^,^14^. *adipoqFAK*^*−/−*^ mice also showed similar results when key experiments were replicated with these animals (Supplementary Fig. 3a–c).

**Figure 2:**
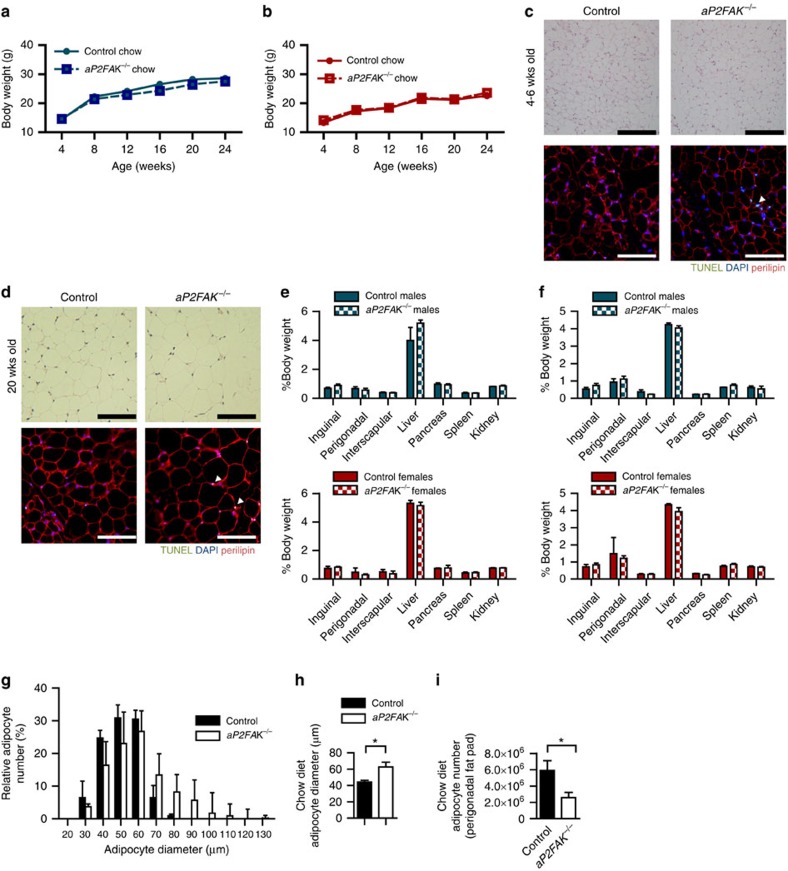
FAK is required for adipocyte survival. (**a**,**b**) Body weight in male (**a**) and female (**b**) littermate control or *aP2FAK*^*−/−*^ mice fed chow diet (*n*=10). (**c**,**d**) Representative haematoxylin and eosin (H&E) and TUNEL of perigonadal WAT sections from 4- to 6-week-old mice (*n*=3 mice) (**c**) and 20-week-old mice (*n*=4 mice) (**d**) (black scale bar, 100 μm; white scale bar, 100 μm, arrows indicate positive nuclei). (**e**,**f**) Body composition expressed as per cent total body weight in 4- to 6-week- (*n*=5) (**e**) and 20-week-old (**f**) male (blue) and female (red) mice (*n*=6 males, 5 females). (**g**–**i**) Adipocyte size distribution (**g**), average adipocyte diameter (**h**) and calculated total adipocyte number (**i**) in 20-week-old mice (*n*=4 mice). Data are mean±s.e.m. *P<0.05 by Student's *t*-test between means.

**Figure 3:**
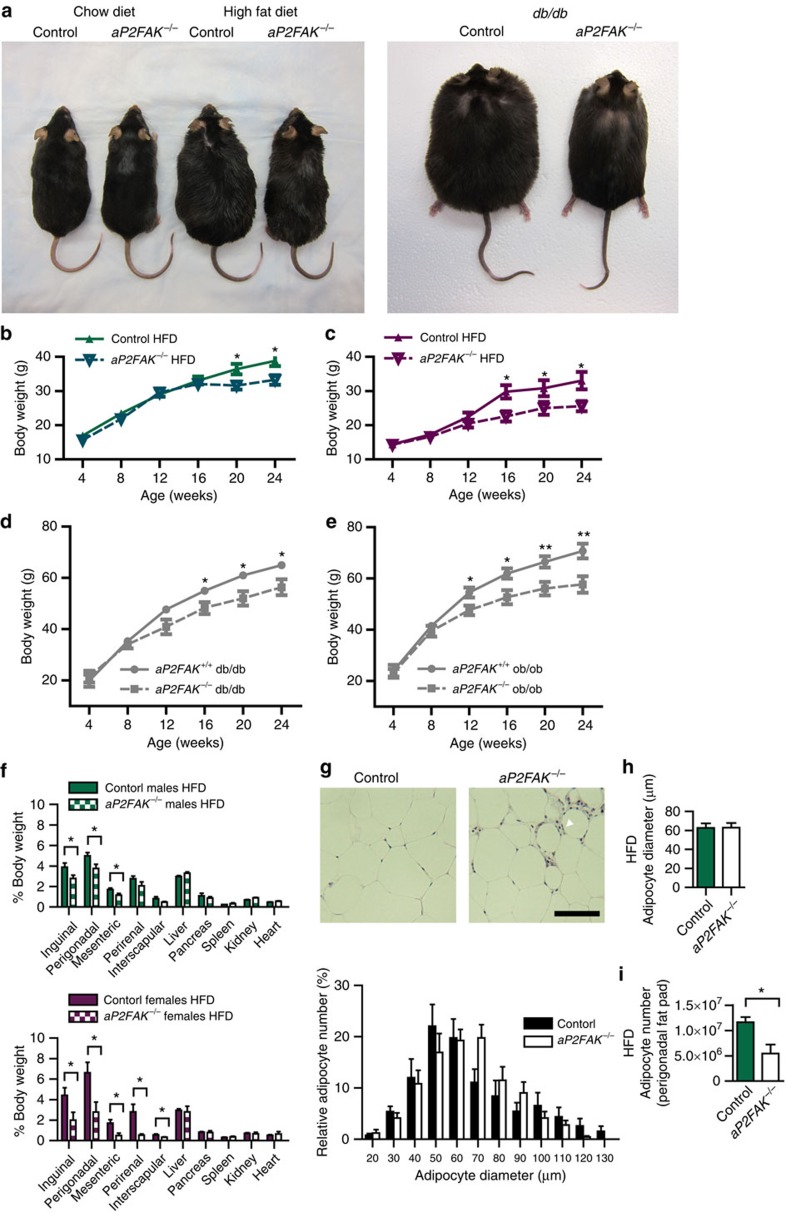
Disruption of FAK impairs adipose tissue expansion with caloric excess. (**a**) Photograph of 24-week-old littermate control and *aP2FAK*^*−/−*^ mouse fed chow or HFD for 16 weeks, and littermate control *aP2FAK*^*+/+*^ *db/db* and *aP2FAK*^*−/−*^ *db/db* mouse. (**b**–**e**) Body weight of male (*n*=12) (**b**) or female (*n*=8) (**c**) mice fed HFD starting at 8 weeks of age or mice on *db/db* (*n*=10) (**d**) or *ob/ob* (*n*=8) (**e**) genetic background. (**f**) Body composition expressed as per cent total body weight in 20- to 24-week-old male (green) and female (purple) mice fed HFD diet for 12–16 weeks (*n*=6). (**g**–**i**) Perigonadal WAT sections stained with haematoxylin and eosin (H&E) (scale bar, 100 μm; arrows indicate macrophage crown-like structures) with adipocyte size distribution (**g**), average adipocyte diameter (**h**) and calculated total adipocyte number (**i**) from 20- to 24-week-old mice fed HFD for 12–16 weeks (*n*=4 mice). Data are mean±s.e.m. *P<0.05 and **P<0.01 by Student's *t*-test between means.

Both male and female *aP2FAK*^*−/−*^ mice and *adipoqFAK*^*−/−*^ mice appeared normal and showed no differences in total body weight or weight gain compared with littermate controls when fed a standard chow diet and followed up to 6 months of age (Figs 2a,b and 3a, and Supplementary Fig. 3b). Animals had no significant difference in nose-to-anus length or femur length (Supplementary Fig. 3a,d,e). However, when we examined levels of apoptosis in adipose tissue by transferase-mediated dUTP nick-end labelling (TUNEL), *aP2FAK*^*−/−*^ mice had increased levels of apoptosis in WAT compared with control littermates starting as early as 4–6 weeks and persisting to 20 weeks of age (Fig. 2c,d). Consistent with this, cleaved caspase 3, a major effector of apoptosis, and other mediators of apoptosis, Fas and Fas ligand, were increased in *aP2FAK*^*−/−*^ mouse adipose tissue, with similar results in different adipose tissue fat pads and no significant difference in proliferation assessed by Ki67 staining (Supplementary Fig. 4a–c). Adipocyte-specific perilipin, which is decreased in dead or dying adipocytes, was also lower in *aP2FAK*^*−/−*^ mice (Supplementary Fig. 5a). This increase in cell death was not seen in other tissues such as liver or muscle as shown by low levels of cleaved caspase 3 and no difference in expression of pro-apoptotic genes p53 upregulated modulator of apoptosis (PUMA) (*Bbc3*) and Bax (*Bax*) (Supplementary Fig. 5b,c).

Under basal conditions, in keeping with their similar total body weight, *aP2FAK*^*−/−*^ and control mice had similar body composition, including subcutaneous inguinal and visceral perigonadal WAT, and interscapular BAT fat pad weights (Fig. 2e,f). However, associated with persistent increase in adipocyte apoptosis, by 20 weeks of age, *aP2FAK*^*−/−*^ mice had a decreased total number of perigonadal fat pad adipocytes as calculated based on adipocyte diameter and fat pad mass, and a compensatory increase in average adipocyte diameter (Fig. 2g–I and Supplementary Fig. 5d,e). Subcutaneous inguinal WAT and interscapular BAT also demonstrated similar histological changes (Supplementary Fig. 5f,g). Together, these findings suggest that FAK is required for ongoing adipocyte survival and disruption of FAK is associated with increased adipocyte apoptosis and decreased estimated adipocyte number.

### Disruption of FAK impairs adipose tissue expansion

To further investigate the consequences of *FAK* knockdown, we studied both male and female *aP2FAK*^*−/−*^ mice in the setting of obesity and insulin resistance induced either by HFD feeding for 12 weeks or on a genetically obese *db/db* or *ob/ob* background. In an environment of energy excess, by 12–16 weeks of HFD, *aP2FAK*^*−/−*^ mice gained less weight than control *aP2FAK*^*+/+*^ mice on HFD (Fig. 3a–c). Similarly, aP2*FAK*^*−/−*^ *db/db* or aP2*FAK*^*−/−*^ *ob/ob* mice gained less weight than control *aP2FAK*^*+/+*^*db/db* or *aP2FAK*^*+/+*^*ob/ob* littermates, respectively (Fig. 3a,d,e).

**Figure 4:**
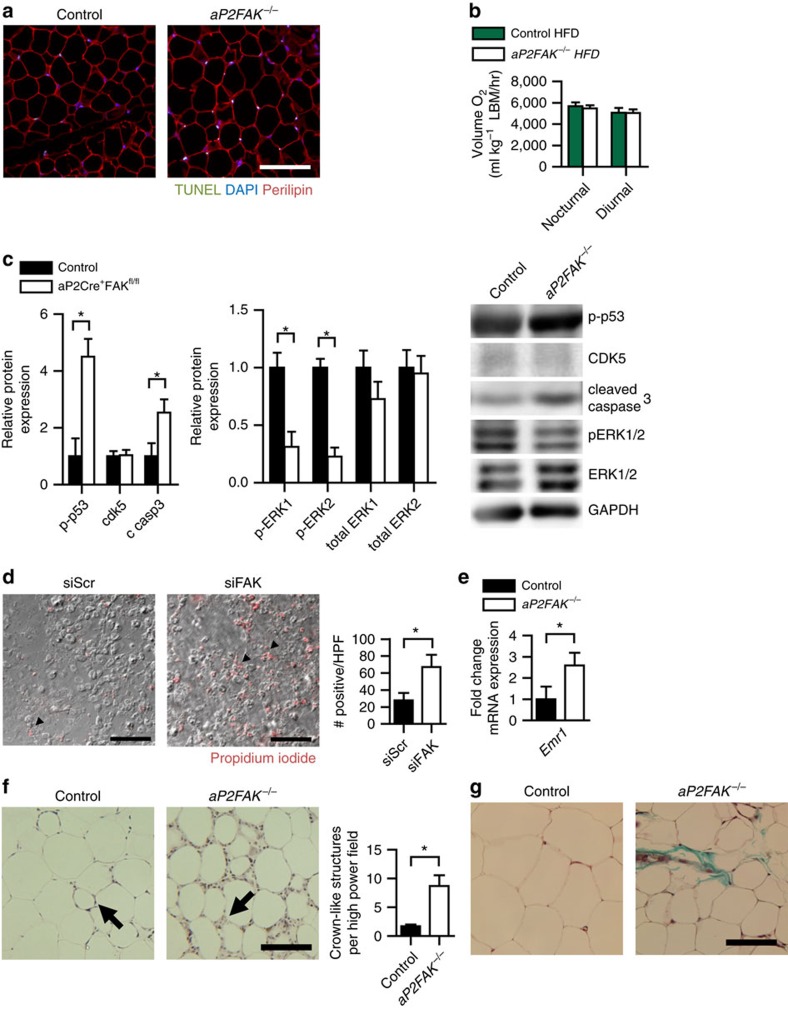
Disruption of FAK decreases survival signalling. (**a**) Representative TUNEL of perigonadal WAT sections from 20- to 24-week-old mice fed HFD for 12–16 weeks (scale bar, 100 μm; arrows indicate TUNEL-positive nuclei) (*n*=4 mice). (**b**) Energy expenditure measured by oxygen consumption normalized to lean body mass (LBM) in mice fed HFD (*n*=3 control or 4 *aP2FAK*^*−/−*^ mice). (**c**) Representative western blotting for cell survival signalling proteins in adipocytes from perigonadal WAT of HFD-fed mice with quantification (*n*=3 mice). (**d**) Representative PI staining of 3T3-L1 adipocytes day 7 after siRNA knockdown of FAK (scale bar, 100 μm; arrows indicate PI-positive nuclei) with quantification (*n*=3 replicates). (**e**) Relative macrophage F4/80 gene (*Emr1*) expression in whole perigonadal WAT of *aP2FAK*^*−/−*^ versus control mice (*n*=6 mice). (**f**,**g**) Macrophage crown-like structures (arrow) seen with haematoxylin and eosin (H&E) staining with quantification (**h**) and fibrosis seen with Masson's trichrome staining (**i**) in perigonadal WAT sections from 24-week-old HFD-fed mice (scale bar, 100 μm). Data are mean±s.e.m. *P<0.05 by Student's *t*-test.

After 12–16 weeks of HFD, decreased weight gain seen in mice with FAK knockdown consisted primarily of decreased fat pad weight for subcutaneous inguinal, visceral perigonadal and mesenteric fat pads compared with littermate controls (Fig. 3f). There was no significant difference in average cell diameter in this setting, but decreased total perigonadal fat pad adipocyte number as estimated based on adipocyte diameter and fat pad weight (Fig. 3g–i). Levels of adipocyte apoptosis, as shown by TUNEL, was increased in WAT of FAK-deficient mice compared with that of control littermates (Fig. 4a). Consistent with this, greater increases were seen in cleaved caspase 3, Fas and Fas ligand, with a decrease in perilipin and similar results in different adipose tissue fat pads (Supplementary Fig. 4a–c).

No significant differences were seen in the weight of other tissues including the liver, pancreas or spleen, and no histological differences were seen in the liver or muscle between HFD-fed *aP2FAK*^*−/−*^ and control mice (Fig. 3f and Supplementary Fig. 5h,i). Measurement of energy expenditure was consistent with body composition findings. In chow diet-fed mice at younger (4–6 weeks) or older (20 weeks) age, with no difference in body weight, there was no difference in energy expenditure, suggesting that disruption of FAK did not directly alter oxygen consumption (Supplementary Fig. 6a,b). With 16 weeks on HFD or on *db/db* background, *aP2FAK*^*−/−*^ mice with lower body weight and decreased adiposity also had similar total levels of oxygen consumption compared with control littermates, suggesting that energy expenditure was consistent with similar lean body mass in the *aP2FAK*^*−/−*^ mice (Supplementary Fig. 6c,d). Furthermore, normalizing oxygen consumption to lean body mass, as determined by magnetic resonance imaging, confirmed that energy expenditure was proportional to lean body mass (Fig. 4b). There was no difference in respiratory exchange ratio, activity level, food or water intake between groups (Supplementary Fig. 6c,d). Overall, these results are in keeping with decreased adipose tissue expansion with FAK deficiency in an environment of energy excess without any primary defects in energy expenditure.

### FAK promotes survival signalling in adipocytes

A key target of FAK is tumour suppressor p53, which FAK has been shown to bind and degrade in fibroblasts, contributing to apoptosis under cellular stress conditions^16^. Disruption of FAK in this setting thereby activates p53 to promote apoptosis and suppress tumour growth via regulation of ERK1/2 signalling^7^,^11^,^12^,^17^,^18^. To determine whether this pathway is also important in adipocytes, we measured levels of phosphorylated p53, which were increased in adipocytes from *aP2FAK*^*−/−*^ mice compared with controls (Fig. 4c and Supplementary Fig. 7a). In some contexts, p53 also arrests cell cycle progression; in mouse adipocytes we saw no change in mRNA levels of cyclin E (*Ccne*) or protein levels of cyclin-dependent kinase 5, a cell cycle regulator thought to act downstream of FAK in some contexts^19^,^20^ (Fig. 4c and Supplementary Fig. 7a,b). *Rb*, which also plays important roles in cell cycle inhibition and promoting apoptosis was increased as shown by mRNA levels, in keeping with increased cell death (Supplementary Fig. 7b). Importantly, in many contexts, FAK activates ERK1/2 to promote cell survival. Accordingly, we measured ERK1/2 phosphorylation, which was decreased following deletion of FAK (Fig. 4c and Supplementary Fig. 4a,b). These results were similar under both chow and HFD conditions (Supplementary Fig. 4a,b). Finally, mRNA levels of a major downstream target of p53 and activator of apoptosis, *Puma*^21^,^22^, but not *Bax*, was increased in adipocytes from mice with FAK deletion (Supplementary Fig. 7b). Ultimately, this was associated with upregulation of apoptosis via cleaved caspase 3 and adipocyte cell death (Fig. 4c and Supplementary Figs 4a,b and 5a).

To further study the cell-autonomous role of FAK in mature adipocytes, we used small interfering RNA (siRNA) to knock down *FAK* in 3T3-L1 adipocytes starting at day 0 of differentiation (Supplementary Fig. 7c,d). Consistent with findings *in vivo*, deletion of *FAK* directly in adipocytes resulted in increased cell death, as shown by propidium iodide staining (Fig. 4d). *Puma* gene expression was also increased, consistent with findings *in vivo* (Supplementary Fig. 7b,e). Apoptosis was upregulated via increased cleaved caspase 3 in 3T3-L1 adipocytes (Supplementary Fig. 7d) and simultaneous knockdown of *FAK* and *p53* or treatment with caspase inhibitor Z-VAD-FMK (ZVAD) prevented increases in apoptosis, in keeping with the essential role of FAK in apoptosis (Supplementary Fig. 7f). In addition, treatment of mature 3T3-L1 adipocytes with FAK inhibitor PF573228 also increased cleaved caspase 3, further supporting a direct pro-survival signalling of FAK in mature adipocytes (Supplementary Fig. 7g). Moreover, treatment of primary isolated adipocytes with PF573228 also resulted in similar findings. Phosphorylated p53 increased and p-ERK1/2 decreased following inhibition of FAK (Supplementary Fig. 8a). *Puma* gene expression and cleaved caspase 3 protein were increased following treatment with FAK inhibitor and treatment with ZVAD prevented increases in apoptosis (Supplementary Fig. 8b,c). These findings further show that in adipocytes, FAK provides an essential pro-survival signal, likely to be via regulation of p53.

With obesity, adipocyte death is thought to be a strong phagocytic stimulus, driving recruitment of macrophages and inflammation in adipose tissue^4^,^5^,^6^,^23^. Under basal conditions, *aP2FAK*^*−/−*^ mice had evidence of increased macrophage infiltration of adipose tissue as shown by macrophage-specific F4/80 gene (*Emr1*) expression and immunofluorescence in whole adipose tissue (Fig. 4e and Supplementary Fig. 8d). With HFD feeding, adipose tissue inflammation-associated macrophage recruitment was markedly more pronounced, with increased numbers of macrophage crown-like structures seen (Fig. 4f). Fibrosis, another consequence of increased inflammation, was also seen to a greater degree with Masson's Trichrome staining in adipose tissue from HFD-fed *aP2FAK*^*−/−*^ mice (Fig. 4g). Overall, this demonstrates that disruption of FAK signalling resulted in increased cell death and adipose tissue inflammation, contributing to metabolic disturbance.

### Adipocyte FAK is required to maintain insulin sensitivity

**Figure 5:**
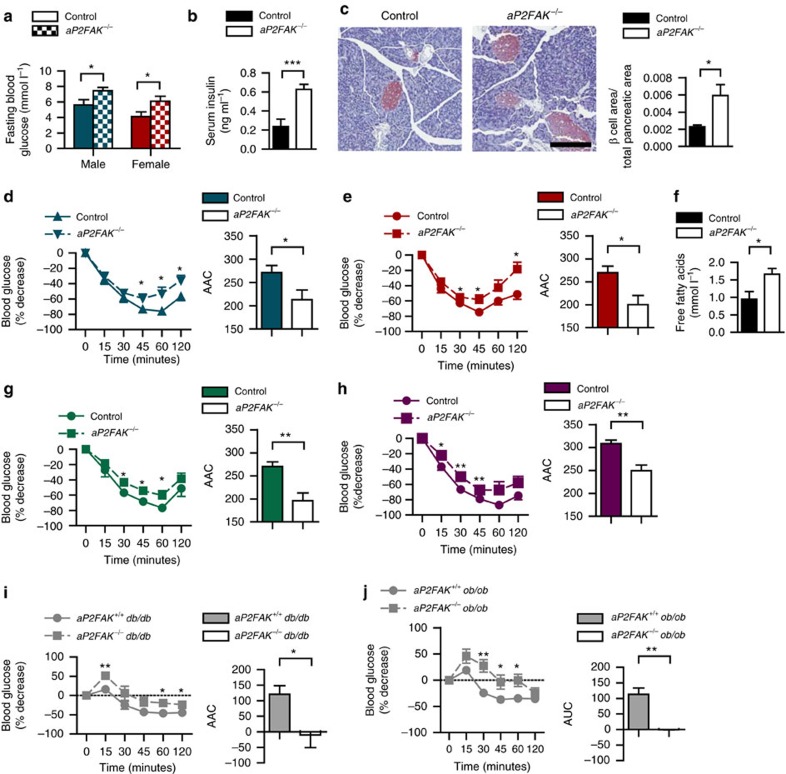
Adipocyte FAK is required for maintenance of glucose homeostasis. (**a**) Fasting blood glucose in male (*n*=9) (blue) and female (*n*=10) (red) control or *aP2FAK*^*−/−*^ mice at 12 weeks of age. Fasting serum insulin (*n*=10) (**b**) and pancreas sections from 20- to 24-week-old mice stained by IHC for insulin (red; scale bar, 200 μm) with quantification (*n*=5 mice) (**c**). ITT and area above the curve (AAC) in male (*n*=12) (**d**) and female (*n*=10) (**e**) 20- to 24-week-old mice. (**f**) Fasting serum free fatty acid levels in 20- to 24-week-old mice (*n*=6). ITT and AUC in male (*n*=10) (**g**) and female (*n*=9) (**h**) 20- to 24-week-old mice fed HFD for 12–16 weeks or 6- to 8-week-old mice on *db/db* (*n*=6) (**i**) or *ob/ob* genetic background (*n*=9) (**j**). Data are mean±s.e.m. * *P*<0.05, **P<0.01 and ***P<0.001 by Student's *t*-test.

Adipose tissue plays a central role in the regulation of insulin sensitivity, as shown by studies demonstrating that disruption of adipocyte function at the molecular level has profound consequences for whole-body glucose metabolism^13^,^14^,^24^. To determine the role of FAK in adipose tissue regulation of whole-body glucose homeostasis, we monitored fasting blood glucose levels in *aP2FAK*^*−/−*^ mice on chow diet. By 12 weeks of age, both male and female *aP2FAK*^*−/−*^ mice had elevated fasting blood glucose compared with littermate controls (Fig. 5a and Supplementary Fig. 9a,b). Disruption of FAK increased insulin resistance in both male and female *aP2FAK*^*−/−*^ mice as seen by elevated fasting serum insulin levels, increased β-cell to pancreas area and insulin tolerance testing (ITT), without changes in glucose tolerance (Fig. 5b–e and Supplementary Fig. 9c,d). In keeping with insulin resistance in adipose tissue, decreased phosphorylation of Akt was seen in response to insulin in perigonadal WAT and interscapular BAT, but not in the liver or muscle of *aP2FAK*^*−/−*^ mice compared with control littermates (Supplementary Fig. 9e). Interestingly, Akt phosphorylation was unchanged in 3T3-L1 adipocytes with direct *FAK* knockdown, suggesting that the improved insulin sensitivity in adipose tissue of *aP2FAK*^*−/−*^ mice was likely to be an indirect effect (Supplementary Fig. 9f). Consistent with impaired metabolic dysfunction, other features, including hyperlipidemia, were present as shown by higher fasting serum free fatty acid and total cholesterol levels in *aP2FAK*^*−/−*^ mice compared with littermate controls (Fig. 5f and Supplementary Fig. 9g,h). Similarly, adipoqFAK^*−/−*^ mice were also more insulin resistant on ITT compared with littermate controls (Supplementary Fig. 3c).

The increase in insulin resistance observed under chow diet conditions in FAK deficiency was also seen with reduced adiposity in the setting of HFD or genetic obesity. This was shown by attenuated glucose lowering during ITT in *aP2FAK*^*−/−*^ mice compared with littermate controls with HFD feeding or on a *db/db* or *ob/ob* background (Fig. 5g–j). No difference in glucose tolerance testing was seen, likely to be due to compensation by pancreatic β-cells (Supplementary Fig. 9i–k). Overall, these results show that adipocyte FAK is required for maintenance of insulin sensitivity under both basal and metabolic stress conditions.

### Adiposity and insulin resistance not restored by PPARγ

**Figure 6:**
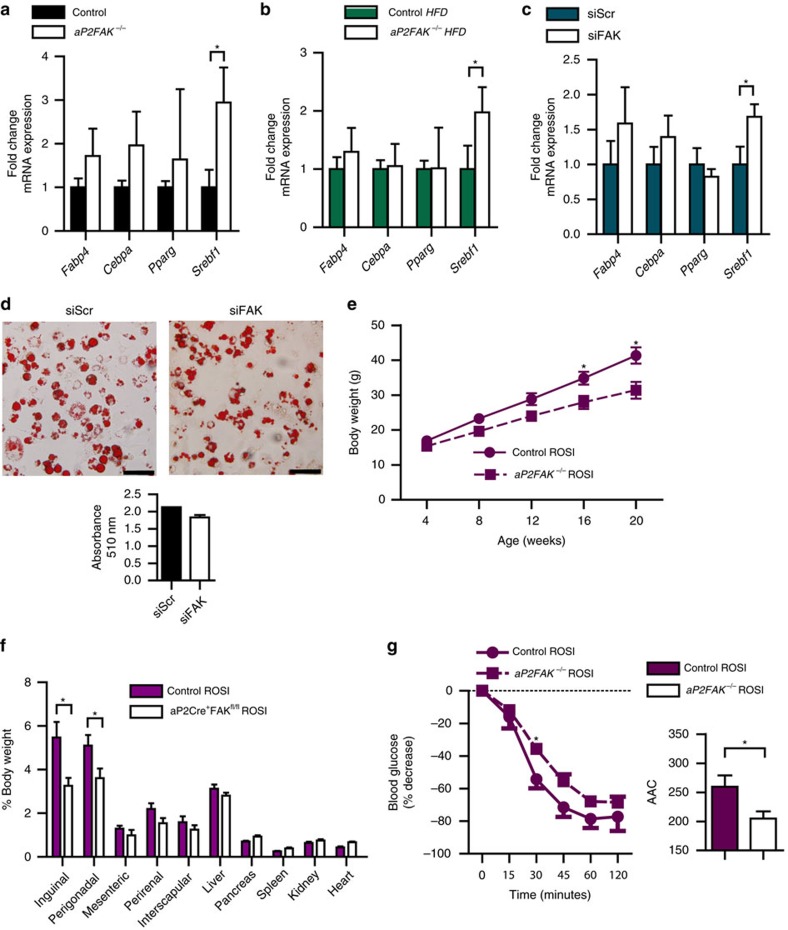
Impaired adipose tissue expansion with disruption of FAK cannot be overcome by PPARγ agonist. (**a**–**c**) Relative expression of adipogenic genes from perigonadal WAT of *aP2FAK*^*−/−*^ versus control 20- to 24-week-old mice fed chow diet (*n*=7) (**a**) or HFD (*n*=5) (**b**) and in 3T3-L1 cells day 3 after FAK versus control scramble siRNA treatment (*n*=6) (**c**). (**d**) Oil red O staining of differentiated 3T3-L1 adipocytes day 7 following siRNA knockdown of FAK (scale bar, 100 μm). (**e**) Body weight in 20- to 24-week-old control or *aP2FAK*^*−/−*^ mice fed HFD with rosiglitazone for 12–16 weeks (*n*=7). (**f**) Body composition expressed as percent total body weight (*n*=10). (**g**) ITT in 20- to 24-week-old mice fed HFD with rosiglitazone for 12–16 weeks (*n*=5). Data are mean±s.e.m. *P<0.05 by Student's *t*-test.

As FAK is also essential for development^7^, we hypothesized that a decrease in adipogenesis could also contribute to the changes in adiposity in these mice. Accordingly, we measured expression of genes involved in adipogenesis in *aP2FAK*^*−/−*^ mice normalized to control littermates and no major differences in adipocyte protein 2 (*Fabp4*), CCAAT/enhancer binding protein-α (*Cebpa*) or peroxisome proliferator-activated receptor-γ (*Pparg*) were observed in either perigonadal or inguinal WAT, under basal conditions or with HFD (Fig. 6a,b and Supplementary Fig. 9l). Similar results were seen in 3T3-L1 adipocytes following siRNA-mediated knockdown of FAK on day 0 of preadipocyte differentiation, which significantly decreases *FAK* gene and protein expression by days 3 to 7 (Fig. 6c and Supplementary Fig. 7c,d) and in isolated primary mouse adipocytes treated with FAK inhibitor PF573228 (Supplementary Fig. 9m). There was an increase in sterol regulatory element-binding transcription factor 1 (SREBP-1c, *Srebf1*) (Fig. 6a–c), which has been associated with disruption of adipocyte biology and lipodystrophy^25^. As SREBP-1c is also associated with lipogenesis^26^,^27^,^28^, we examined expression of other genes involved in lipogenesis or lipolysis and did not see major differences in their expression following disruption of FAK *in vivo* (Supplementary Fig. 9n). SREBP-1c has also been hypothesized to regulate adipogenic gene *Pref1* (ref. 25), but we saw no differences in *Pref1* expression (Supplementary Fig. 9n). In support of the lack of changes in adipogenesis, oil red O (ORO) staining did not show significant differences following 3T3-L1 adipocyte differentiation with FAK knockdown (Fig. 6d).

Finally, PPARγ agonists are well known for increasing adipocyte mass and improving insulin sensitivity. To determine whether impairment of adipose tissue expansion and insulin resistance could be overcome by a PPARγ agonist, *aP2FAK*^*−/−*^ and control mice were fed HFD with rosiglitazone. Even when treated with rosiglitazone, *aP2FAK*^*−/−*^ mice failed to gain as much weight on HFD as littermate controls and had decreased adipose tissue mass (Fig. 6e,f). *aP2FAK*^*−/−*^ mice also remained more insulin resistant than controls (Fig. 6g). These data suggest that FAK is required for adipose tissue expansion and maintenance of glucose homeostasis, including in response to PPARγ agonist, and rosiglitazone was insufficient to overcome the defect present in *aP2FAK*^*−/−*^ mice.

### Inhibiting apoptosis restores adiposity and insulin response

**Figure 7:**
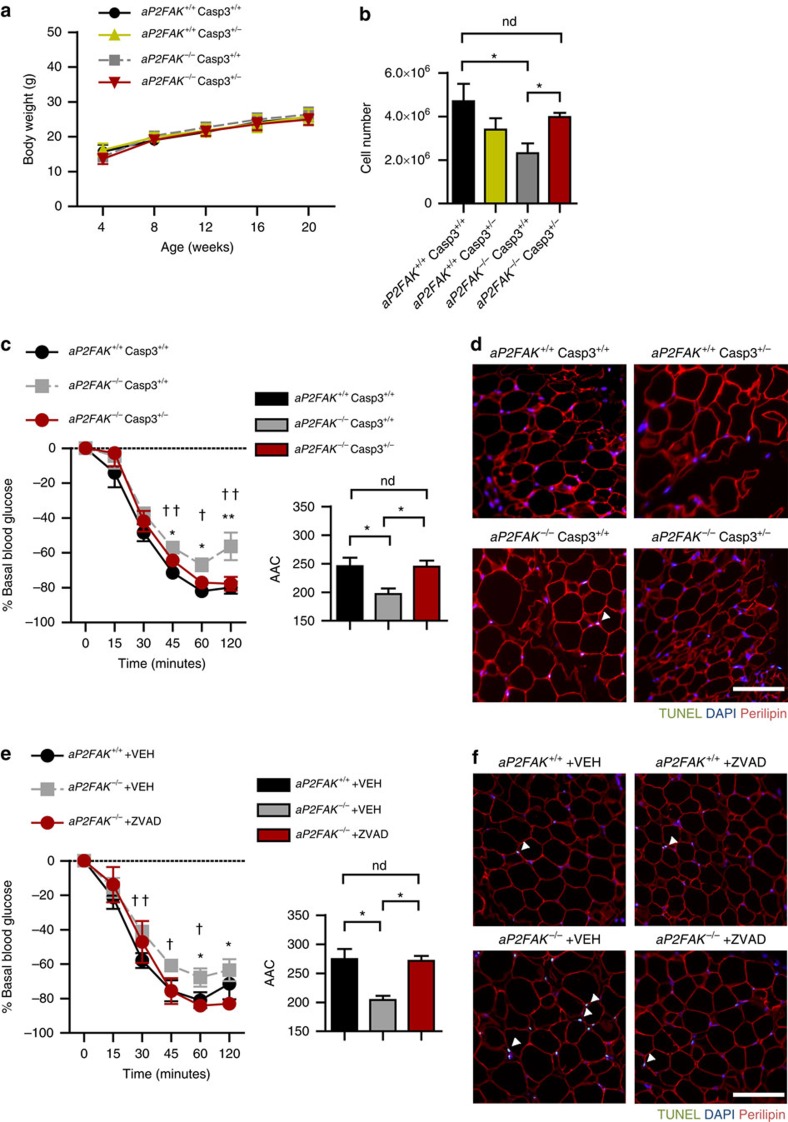
Inhibiting apoptosis restores adiposity and insulin sensitivity with FAK deletion. (**a**) Body weight in littermate control or *aP2FAK*^*−/−*^ *Casp3*^*+/−*^ mice fed chow diet (*n*=6). (**b**) Calculated total adipocyte cell number from perigonadal WAT of *aP2FAK*^*−/−*^ *Casp3*^*+/−*^ mice (*n*=4 mice). (**c**) ITT in 20- to 24-week-old *aP2FAK*^*+/+*^ *Casp3*^*+/+*^, *aP2FAK*^*−/−*^ *Casp3*^*+/+*^ and *aP2FAK*^*−/−*^ *Casp3*^*+/−*^ mice and area above the curve (AAC) (*n*=10). *P<0.05 *aP2FAK*^*−/−*^ *Casp3*^*+/−*^ versus *aP2FAK*^*−/−*^ *Casp3*^*+/+*^mice. †*P*<0.05 *aP2FAK*^*−/−*^ *Casp3*^*+/+*^ versus *aP2FAK*^*+/+*^ *Casp3*^*+/+*^ mice. (**d**) Representative TUNEL of perigonadal WAT sections from 20- to 24-week-old *aP2FAK*^*−/−*^ *Casp3*^*+/−*^ mice (scale bar, 100 μm; arrows indicate positive nuclei) (*n*=3 mice). (**e**) ITT and AUC for 20- to 24-week-old *aP2FAK*^*−/−*^ mice treated with Z-VAD-FMK (ZVAD) or vehicle (VEH) and *aP2FAK*^*+/+*^ mice treated with VEH (*n*=3). *P<0.05 *aP2FAK*^*−/−*^ +ZVAD versus *aP2FAK*^*−/−*^ +VEH. †*P*<0.05 *aP2FAK*^*−/−*^ *+*VEH versus *aP2FAK*^*+/+*^+VEH. (**f**) Representative TUNEL of perigonadal WAT sections from 20- to 24-week-old *aP2FAK*^*−/−*^ and *aP2FAK*^*+/+*^ mice fed HFD for 12–16 weeks then treated with ZVAD or VEH (scale bar, 100 μm; arrows indicate positive nuclei) (*n*=3 mice). Data are mean±s.e.m. *P<0.05 by Student's *t*-test. ND, no stastistically significant difference, *P*>0.05 by Student's *t*-test.

Finally, to definitively address the hypothesis that apoptosis is the primary causal defect in *aP2FAK*^*−/−*^ mice responsible for their impaired adipose tissue expansion and increased insulin resistance, we cross-bred them to apoptosis-deficient *Casp3*^*+/−*^ mice, which we have previously generated and characterized^29^. Under chow-fed conditions, *aP2FAK*^*+/+*^ *Casp3*^*+/+*^, *aP2FAK*^*−/−*^ *Casp3*^*+/−*^ and *aP2FAK*^*−/−*^ *Casp3*^*+/+*^ mice had no differences in total body weight (Fig. 7a). Estimated number of adipocytes in the perigonadal fat pad was similar in *aP2FAK*^*+/+*^ *Casp3*^*+/−*^ compared with *aP2FAK*^*+/+*^ *Casp3*^*+/+*^ controls. Remarkably, the decrease in adipocyte number present in *aP2FAK*^*−/−*^ was restored in caspase 3-deficient aP2FAK^*−/−*^ *Casp3*^*+/−*^ mice (Fig. 7b). Moreover, insulin sensitivity as assessed by ITT was improved in *aP2FAK*^*−/−*^ *Casp3*^*+/−*^ mice compared with *aP2FAK*^*−/−*^ *Casp3*^*+/+*^ mice (Fig. 7c and Supplementary Fig. 10a). Finally, the increased apoptosis measured by TUNEL seen in *aP2FAK*^*−/−*^ *Casp3*^*+/+*^ mice was abolished in *aP2FAK*^*−/−*^ *Casp3*^*+/−*^ mice (Fig. 7d). Similarly, improved insulin sensitivity was seen with complete deletion of caspase 3 (*Casp3*) and in mice with adipocyte-specific deletion of caspase 8 (*Casp8*) when compared with littermate controls (Supplementary Fig. 10b,c). Furthermore, with *Casp3* haploinsufficiency, weight gain on HFD was restored in *aP2FAK*^*−/−*^ mice with comparable adiposity as controls (Supplementary Fig. 10e,f). Insulin sensitivity in *aP2FAK*^*−/−*^ *Casp3*^*+/−*^ mice on HFD was also improved compared with *aP2FAK*^*−/−*^ *Casp3*^*+/+*^ mice (Supplementary Fig. 10d). Finally, HFD-fed *aP2FAK*^*−/−*^ *Casp3*^*+/−*^ had similar energy expenditure as controls (Supplementary Fig. 10g).

Moreover, to further determine whether acute inhibition of adipocyte apoptosis might improve insulin sensitivity, we treated HFD-fed *aP2FAK*^*−/−*^ mice with caspase inhibitor ZVAD and performed ITT 24 h later, to assess insulin sensitivity. Remarkably, acute inhibition of apoptosis partially reduced TUNEL in perigonadal WAT and improved insulin sensitivity (Fig. 7e,f). Similarly, *in vitro*, ZVAD treatment of 3T3-L1 adipocytes with FAK knockdown or primary adipocytes treated with FAK inhibitor PF573228 reduced levels of cleaved caspase 3, consistent with a direct role of FAK in mediating cell death (Supplementary Figs 7f and 8c). Together, using genetic or pharmacologic approaches *in vivo* and *in vitro*, we show a causal role for apoptosis in promoting insulin resistance following adipocyte deletion of *FAK*. These data further support a key role of FAK in preventing adipocyte cell death due to metabolic stress, thereby maintaining insulin sensitivity.

## Discussion

In this study, we find that the major intracellular signalling node FAK plays an essential role in adipose tissue, particularly in response to metabolic stress. FAK is induced in adipose tissue with metabolic stress from caloric excess. We find that disruption of FAK impairs adipocyte survival, increasing activation of p53 and decreasing phosphorylation of ERK, resulting in decreased cell survival *in vitro* and *in vivo*. Ultimately, in novel adipocyte-specific *FAK* knockout mice and multiple models of obesity and insulin resistance, decreased FAK signalling reduces adipocyte number and impairs adipose tissue expansion following prolonged caloric excess. Adipocyte FAK is required for maintenance of insulin sensitivity under both basal and metabolic stress conditions. Finally, inhibiting apoptosis genetically or acutely through pharmacologic means restores adipocyte survival and insulin sensitivity. Altogether, these data demonstrate that FAK is required for adipocyte survival and response to metabolic stress.

Studies of FAK to date have mostly focused on its expression in malignancies. Following its discovery in 1992, FAK was found to be overexpressed in a number of human tumours, particularly with invasive or advanced disease^30^,^31^. We therefore sought to identify the role of FAK in metabolic disease and found that it is similarly altered in tissue growth induced by fuel excess, adipose tissue being the most dynamic organ in this context and the focus of this work.

FAK is best recognized for its central role in integrin signalling, which responds to stimuli from the ECM. Therefore, this work builds on previous studies showing the importance of the ECM in obesity and diabetes. Deficiency of key ECM components such as collagen Vα3, matrix metalloproteinase 14 or tissue inhibitor of matrix metalloproteinase 2 can reduce adiposity and impair glucose homeostasis *in vivo*^32^,^33^,^34^,^35^. *In vitro*, ECM stiffness has been shown to regulate adipogenesis and reduction in ECM fibronectin and α5 integrin, which binds fibronectin, facilitates differentiation^36^,^37^,^38^. FAK has been noted to undergo cleavage^39^ and be required for early adipogenesis in cell culture^40^; in contrast, treatment of human mesenchymal stem cells with a FAK inhibitor promoted adipogenesis^41^. The physiological and metabolic consequences of these findings, particularly *in vivo* in mature adipocytes were unclear. Our data did not show major differences in adiposity under basal conditions or changes in adipogenic gene expression with disruption of *FAK* in mature adipocytes. This suggests that FAK may not be essential for adipose tissue development or adipogenesis *in vivo*, although our models result in deletion of *FAK* primarily in mature adipocytes, rather than preadipocytes or stem cells and the role of FAK is likely to be context dependent^13^. Thus, our work sought to define the further essential roles of FAK in mature adipocytes and particularly *in vivo*.

FAK does appear to have a key role in adiposity with increased caloric intake, with disruption of *FAK* resulting in impaired adipose tissue expansion. Although decreased adipocyte survival and numbers were also seen under basal chow diet-fed conditions, differences were probably too small to result in changes in total body weight or composition. Adipocyte size was increased in mice fed a chow diet but not HFD, suggesting a context-specific role for FAK in adipocytes. Under conditions with less metabolic stress, adipocytes may be able to compensate for reduced numbers with hypertrophy. HFD may also result in increased cell size to an extent similar to adipocytes with FAK disruption. Overall, these findings complement findings in mice lacking collagen VI^42^, which also have a context-specific phenotype that is diametrically opposite to *aP2FAK*^*−/−*^ mice in terms of body composition. *col6KOob/ob* mice eventually gain massive weight, while demonstrating improved glucose homeostasis and dampened inflammatory profile. Weakening of the adipose tissue extracellular scaffold is hypothesized to allow for stress-free adipocyte expansion and attenuation in inflammation with improved metabolic function. With metabolic stress, *aP2FAK*^*−/−*^ mice exhibit increased fibrosis, impaired adipose tissue expansion and increased adipose tissue inflammation contributing to insulin resistance. Together, our study illustrates that FAK is critical for the optimal signalling intracellularly from ECM for normal adipose tissue growth and maintenance of insulin sensitivity.

FAK plays an important role in promoting pro-survival signalling^43^,^44^,^45^. Disruption of *FAK* was initially noted to activate pro-apoptotic p53 in endothelial cells during embryogenesis^7^ and interaction between FAK and p53 is a fundamental link between FAK and cell survival signalling^11^,^12^. In fibroblasts, FAK binds to the negative regulator of p53, MDM2, thereby directly promoting its ubiquitination and degradation^16^. FAK also functions as a scaffolding protein to promote cell survival signalling and activates the ERK/MAPK cascade, which promotes tumour growth^12^,^16^,^18^,^46^. In this study, we find that disruption or inhibition of FAK in adipocytes *in vivo* or *in vitro* increases phosphorylated p53 to promote apoptosis. The major downstream target of p53, *Puma*^21^,^22^ is also increased *in vivo* and *in vitro* following *FAK* disruption. Furthermore, disruption of *FAK* decreases ERK1/2 phosphorylation, decreasing cell viability, similar to findings we reported in pancreatic β-cells^20^. Overall, our data suggest that FAK in adipocytes plays a major role in promoting cell survival.

Of particular significance to obesity-associated insulin resistance, adipocyte death has been hypothesized to be the initiating factor in adipose tissue inflammation, leading to massive infiltration of macrophages and systemic insulin resistance^4^,^5^,^6^,^23^. This remains an area of controversy, however, with the precise links between adipocyte death, inflammation and insulin resistance unclear. For example, Feng *et al*.^4^ show that adipocyte cell death and adipose tissue inflammation with metabolic stress can be disassociated. Our work suggests that HFD-induced FAK promotes cell survival and its disruption contributes to both cell death and inflammation, suggesting that a number of pathways are involved in HFD-mediated insulin resistance. We find that disruption of *FAK* results in increased adipocyte cell death both *in vivo* and *in vitro*, and results in increased adipose tissue inflammation. Although disruption of *FAK* increases macrophage infiltration with HFD feeding, the precise interaction between adipocyte FAK and immune cell mediation of adipose tissue inflammation requires further study. We find that this increase in cell death in FAK-deficient mice occurs before the development of significant changes in body composition or insulin sensitivity, identifying it as an early factor in the pathogenesis of insulin resistance. Inhibiting apoptosis, with genetic caspase 3 or caspase 8 deficiency, or short-term treatment with an apoptosis inhibitor, restores adiposity and insulin sensitivity in these mice.

In summary, we show that FAK is increased in adipose tissue on metabolic stress and plays an important role in maintaining adipocyte survival and response to insulin. Disruption of *FAK* increases cell death, contributing to adipocyte depletion and metabolic dysfunction. This work demonstrates a novel role for FAK as an important connection in adipocyte signalling and provides insight in its role in whole body physiology. This identifies FAK as a new molecular link between obesity and insulin resistance.

## Methods

### Animals

Mice with adipocyte-specific deletion of FAK were generated by breeding *aP2Cre*^*+*^ mice^13^ (Jackson Laboratory) with *FAK*^*fl/fl*^ mice (ref. 47) to generate *aP2FAK*^*+/−*^ mice, which were then intercrossed to generate *aP2FAK*^*−/−*^ mice^20^. Genotypes were identified by PCR of ear clip DNA (Supplementary Fig. 11a)^48^,^49^. No differences in body morphology or fasting blood glucose were seen between *aP2Cre*^−^, *aP2FAK*^*+/−*^ and *aP2FAK*^*+/+*^ mice, and littermate *aP2FAK*^*+/+*^ mice were primarily used as controls (Supplementary Fig. 11b–e). *adipoqCre*^*+*^ mice^15^ (Jackson Laboratory) and *Casp8*^*fl/fl*^ mice^50^,^51^ were also used as indicated. *aP2FAK*^*+/−*^ mice were also interbred with *+/db,* +/*ob* (Jackson Laboratory) and *Casp3*^*+/−*^ (ref. 29) mice, to generate *aP2FAK*^*+/−*^ *+/db*, *aP2FAK*^*+/−*^ *+/ob* and *aP2FAK*^*+/−*^ *Casp3*^*+/−*^ mice, which were then intercrossed to generate: *aP2FAK*^*−/−*^ *db/db* and control *aP2FAK*^*+/+*^ *db/db* mice, *aP2FAK*^*−/−*^ *ob/ob* and control *aP2FAK*^*+/+*^ *ob/ob* mice and *aP2FAK*^*−/−*^ *Casp3*^*+/−*^ or *aP2FAK*^*−/−*^ *Casp3*^*−/−*^ with control genotypes as specified. All strains were developed on a C57BL/6 background. Mice were housed in a pathogen-free animal facility with a 12 h light–dark cycle and fed standard irradiated rodent chow ad libitum (5% fat; Harlan Tekad). Where not otherwise specified, both male and female mice were used in equal numbers and no significant differences by sex found. Sample size was estimated based on previous studies using mouse models^52^,^53^,^54^. Animals were excluded if injured or sick, or if glucose or insulin did not inject correctly, which did not occur at a higher frequency in different experimental groups. Animals were randomly assigned to groups by the experimenter; no formal blinding was used. A cohort of mice was fed a HFD (60% fat, 24% carbohydrates and 16% protein based on caloric content; F3282; Bio-Serv) for 12–16 weeks starting at 8 weeks of age. Another cohort of mice was fed a HFD with rosiglitazone 3 mg kg^−1^ per day for 12–16 weeks, a dose previously used in mice^55^. Rosiglitazone dose was confirmed by measuring average food intake and improved glucose tolerance following treatment compared with animals on HFD alone. Z-VAD-FMK (Calbiochem) was administered intraperitoneally 6 mg kg^−1^, a dose previously used in mice^56^. All animal experimental protocols were approved by the Toronto General Research Institute Animal Care Committee.

### *In vivo* metabolic studies

Glucose tolerance tests were performed on animals fasted overnight, 14–16 h, using glucose 1 g kg^−1^ body weight injected intraperitoneally. Insulin tolerance tests were performed on animals fasted for 4 h, using insulin lispro (Lilly) 1.0 U per kg body weight for chow or HFD-fed mice and 1.5 U kg^−1^ for mice on a *db/db* or *ob/ob* background. Measurements of blood glucose were taken at 0, 15, 30, 45, 60 and 120 min after the injection. For energy expenditure measurements, mice were individually housed in metabolic cages with free access to food and water. After 24 h acclimation to the apparatus, data for 24 h were collected and analysed using a comprehensive lab animal monitoring system (Columbus Instruments)^57^. Food and water consumption were determined by weighing food or measuring water volume before and after 24 h. Insulin levels were measured by a mouse insulin ELISA kit (CrystalChem, Inc.). Serum adipokines were measured by luminex technology using a mouse serum adipokine kit (Millipore).

### Body composition

Lean body mass of HFD-fed mice was determined by magnetic resonance imaging (Biospec 70/30; Bruker, Ettlingen, Germany) and semi-automated and manual segmentation tools using MIPAV software^52^,^58^. Total body and fat mass were measured and used to calculate lean body mass.

### Adipocytes

Where indicated, adipocytes were isolated from mouse fat pads^57^,^59^. For primary adipocyte experiments, inguinal fat was used. Specificity of adipocyte isolation was confirmed by quantitative PCR for adipocyte-specific adiponectin (*Adipoq*), macrophage-specific F4/80 (*Emr1*) and genes preferentially expressed in adipocytes (*Srebpf, Lep*) (Supplementary Fig. 11f,g and Supplementary Table 1). For primary adipocyte experiments, the inguinal fat pad was removed and minced with a razor blade. Five hundred microlitres of minced cells were added to 1,000 μl of 1 mg ml^−1^ type 1 collagenase (Worthington) solution prepared in KRBH buffer then incubated at 37 °C with shaking for 30 min. Cell suspension was then centrifuged at 500 *g* for 10 min and the adipocyte fraction transferred to 500 μl warm KRBH buffer. For primary adipocyte experiments, adipocytes were incubated with 10 μM PF573288 (Sigma-Aldrich) and/or 50 μM ZVAD (Millipore), or an equivalent concentration of the dimethyl sulfoxide vehicle used to dissolve PF573228 and/or ZVAD stock solutions, and incubated for at least 1 h at 37 °C before collection of protein or RNA. Total protein extract from cultured human adipocytes were obtained from Zen-Bio. Samples were from healthy women with or without diabetes (*n*=3; average body mass index: 39.5 kg m^−2^, range: 26.1–52.1, average age: 38 years, range: 37–40 for people without diabetes and *n*=4, average body mass index: 45.7 kg m^−2^, range: 39.5–57.4, average age: 47 years, range 40–57 for people with type 2 diabetes).

### Cell culture and imaging

3T3-L1 preadipocyte cells and cell culture reagents were obtained from Zen-Bio. The cell line was authenticated by quality control staining and tested for mycoplasma by vendor. Cells were cultured and differentiated as per the manufacturer's instructions. Cells were transfected with either *FAK*, *p53* or control scramble Silencer Select siRNA (Ambion) with Lipofectamine RNAiMAX transfection reagent (Invitrogen) according to the manufacturer's forward transfection protocol on day 0 of induction of differentiation^60^. Cells were washed with PBS and stained with membrane impermeant dye propidium iodide for 10 min at room temperature^60^. Fluorescent images were obtained by Olympus IX71 microscope configured for phase contrast microscopy and fluorescence imaging with a QImaging Retiga EXi camera and Micromanager 1.3 software (MMstudio Version 1.3.37). For ORO staining, cells were fixed in 4% paraformaldehyde in 0.1 M PBS pH 7.4 for 1 h, then rinsed with isopropanol. Cells were incubated in ORO working solution for 1 h, then rinsed with PBS. Stained cells were incubated with isopropanol for 5 min and absorbance measured at 510 nm. *In vitro* experiments were repeated three to four times.

### Western blotting

Insulin stimulation *in vivo* was performed with 5 units per kg insulin injected intraperitoneally. Tissues were harvested 10 min after injection. Protein lysates were separated by SDS–PAGE and immunoblotted with antibodies for FAK (sc-557), Fas (sc-1023), Fas ligand (sc-834-G), cyclin-dependent kinase 55 (sc-173), cleaved caspase 3 (a-277) (Santa Cruz Biotechnology, Inc.), Akt (4691), phospho-Akt (Ser473) (9271), phospho-ERK1/2 (9101), perilipin (3470), phospho-p53 (12571), p53 (2524S), glyceraldehydes-3-phosphate dehydrogenase (2118) and phospho-FAK (Tyr397) (3283) (Cell Signaling). Primary antibodies were diluted 1:1,000 and secondary antibodies diluted 1:3,000. Band intensities were quantified using ImageJ software^61^. Uncropped images of Western blots are available in Supplementary Fig. 12.

### RNA isolation and quantitative real-time PCR

Adipocyte RNA was isolated using TRIzol reagent (Invitrogen). RNA was reverse transcribed by random primers using M-MLV (Invitrogen) and quantitative reverse transcriptase–PCR was performed with 7900HT Fast-Real Time PCR System (Applied Biosystem) with SYBR Green master mix reagent (Applied Biosystem). Primers used are listed in Supplementary Table 1. The expression level of each test gene was normalized to the internal control 18s (*Rn18S*). Each sample was run in triplicate^52^.

### Histology

Adipose, pancreas, liver and muscle tissues were harvested after an overnight fast and fixed in 4% paraformaldehyde in 0.1 M PBS (pH 7.4). Sections were stained with haematoxylin and eosin. Perigonadal adipose tissue sections were used for TUNEL (Roche Biochemicals) and immunofluorescence for perilipin (Cell Signaling) and F4/80 (Santa Cruz Biotechnology, Inc.). For cell size and TUNEL analysis, at least 100 cells were counted per mouse. Adipocyte size was measured using ImageJ software. Number of adipocytes in the perigonadal fat pad was calculated from adipocyte diameter and fat pad mass^52^,^62^. Macrophages were excluded from adipocyte size and number calculations by appearance in crown-like structures or positive staining for F4/80 (refs 54, 59). Pancreatic sections were immunostained for insulin and scanned by a ScanScope ImageScope system at × 20 magnification. β-Cell area was quantified using Image Scope software (Aperio Technologies)^20^.

### Statistics

Data are presented as mean±s.e.m. and was analysed by two-tailed independent-sample Student's *t*-test for comparisons between two groups. Two-way analysis of variance was used for multiple measurements as appropriate. *P*-values<0.05 were considered as statistically significant.

### Data availability

The authors declare that the data supporting the findings of this study are available within the paper and its Supplementary Information files, or are available from the corresponding author upon reasonable request.

## Additional information

**How to cite this article:** Luk, C. T. *et al*. FAK signalling controls insulin sensitivity through regulation of adipocyte survival. *Nat. Commun.* **8,** 14360 doi: 10.1038/ncomms14360 (2017).

**Publisher's note**: Springer Nature remains neutral with regard to jurisdictional claims in published maps and institutional affiliations.

## Supplementary Material

Supplementary Information Supplementary Figures, Supplementary Table.
