# Supplementary material for: FAK signalling controls insulin sensitivity through regulation of adipocyte survival
Source: Nat Commun. 2017 Feb 6;8:14360. doi: 10.1038/ncomms14360 (PMC5303880; doi:10.1038/ncomms14360)
Supplement: Supplementary Information — Supplementary Figures, Supplementary Table. [file ncomms14360-s1.pdf]

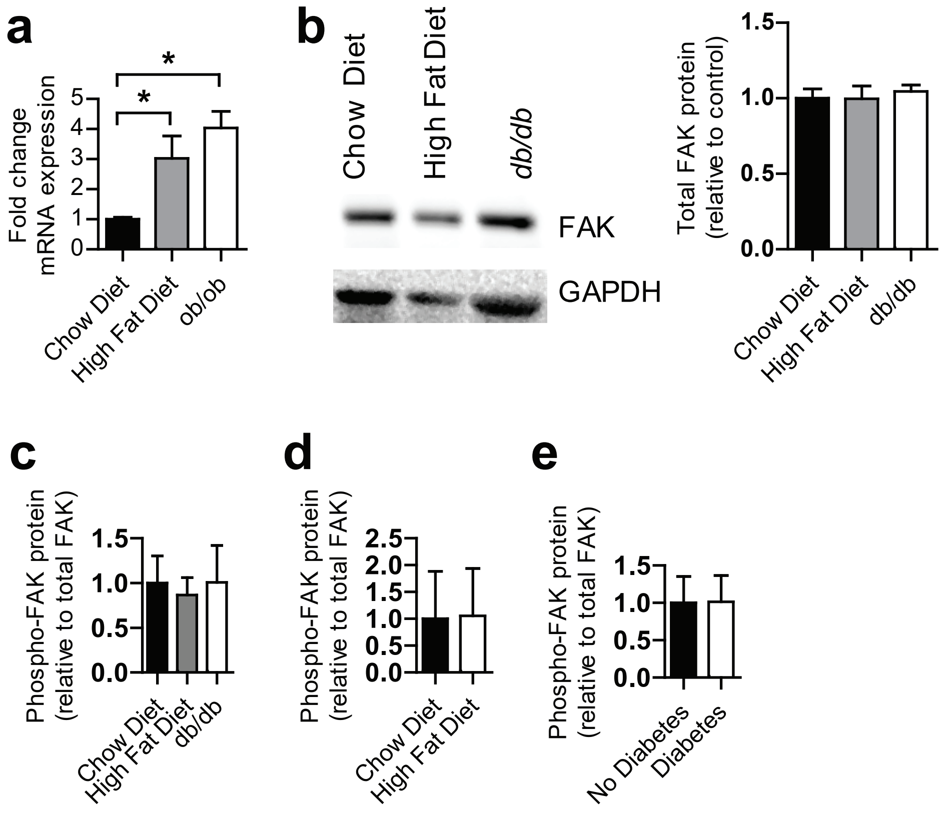

Supplementary Figure 1. FAK phosphorylation in adipose tissue. (**a**) Relative FAK gene (*Ptk2*) expression in adipocytes isolated from mice fed HFD for 12 weeks or db/db mice relative to chow diet-fed mice at 20 weeks old (n = 3 mice). (**b**) Representative blot and quantification of FAK protein in stromal vascular cells from perigonadal white adipose tissue of mice fed high fat diet (HFD) or *db/db* mice relative to chow diet-fed mice (n = 3 mice). (**c-e**) Relative protein levels of phospho-FAK (Tyr397) in adipocytes from perigonadal adipose tissue (**c**) and interscapular brown adipose tissue (**d**) of mice fed HFD or *db/db* mice relative to chow diet-fed mice (n = 3 mice), and in human omental adipocytes (n = 3 humans) (**e**) by densitometric analysis. Data are mean ± SEM. * $p < 0.05$ by Student's t-test.

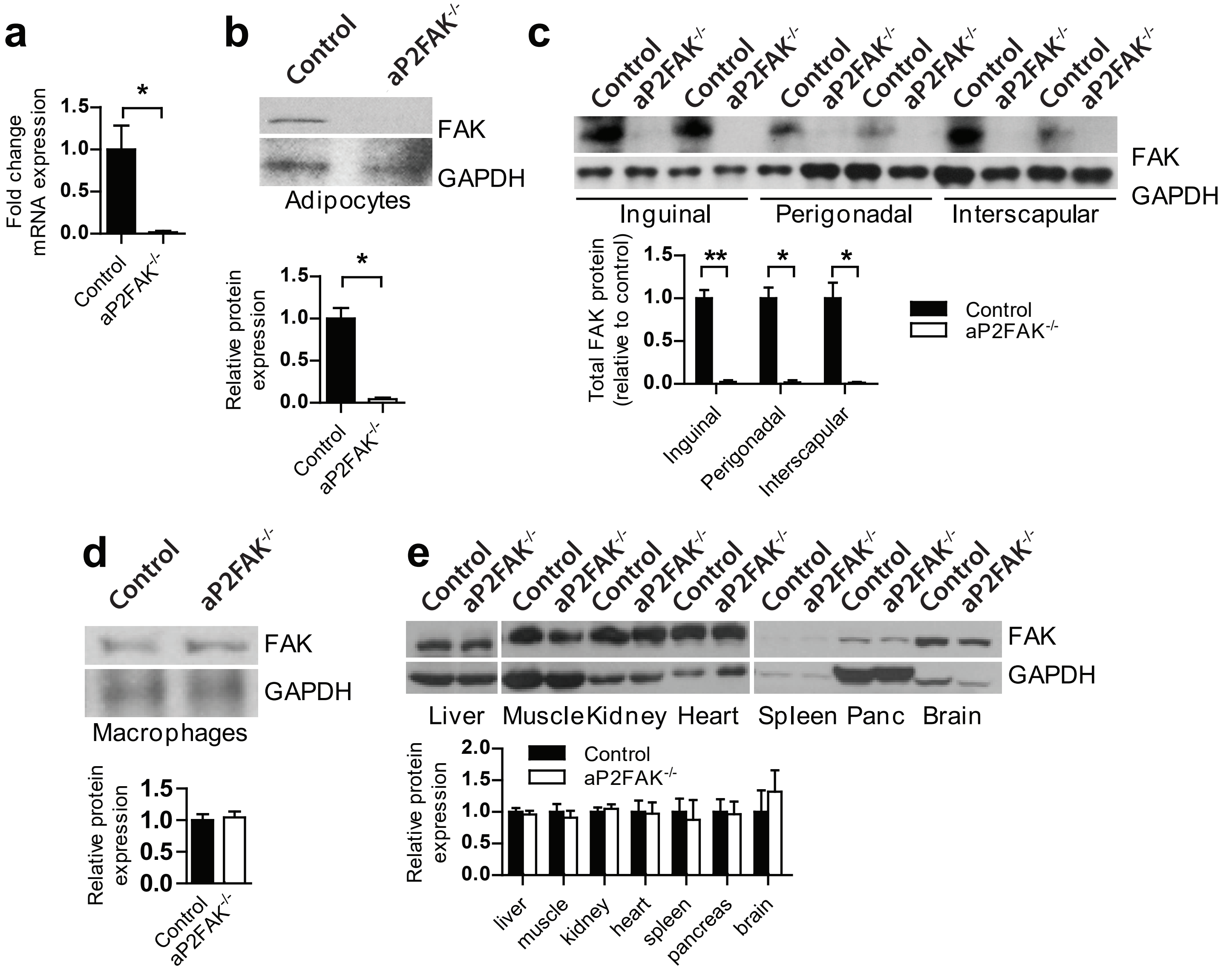

Supplementary Figure 2. FAK in adipose tissue of *aP2FAK$^{-/-}$* mice. (**a**) Relative FAK gene (*Ptk2*) expression in adipocytes isolated from perigonadal fat pads from aP2FAK$^{-/-}$ versus littermate control mice (n = 4 mice). (**b-e**) Representative Western blot of FAK in adipocytes isolated from perigonadal fat pads (**b**), adipose tissue fat pads (**c**), peritoneal macrophages (**d**), and other tissues (**e**) (n = 3 mice). (Pancreas, Panc). Data are mean ± SEM. * $p < 0.05$, ** $p < 0.01$ by Student's t-test.

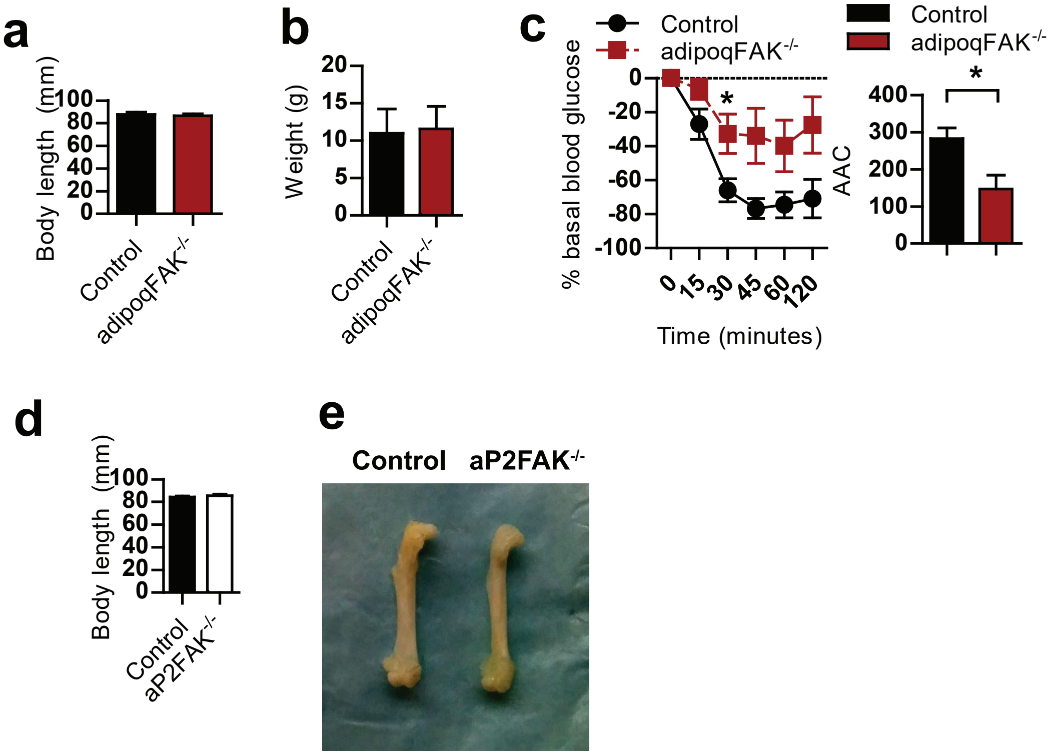

Supplementary Figure 3. ***adipoqFAK***$^{-/-}$ **mice length, weight and insulin sensitivity**. (**a-c**) Nose to anus body length (**a**), weight (**b**) and insulin tolerance testing (**c**) in 6 week old *adipoqFAK*$^{-/-}$ or littermate control mice (n = 5). (**d**) Nose to anus body length in 20-24 week old *aP2FAK*$^{-/-}$ or littermate control mice (n = 9). (**e**) Photograph of femur from 20-24 week old *aP2FAK*$^{-/-}$ and control mouse. Data are mean ± SEM. * $p < 0.05$ by Student's t-test.

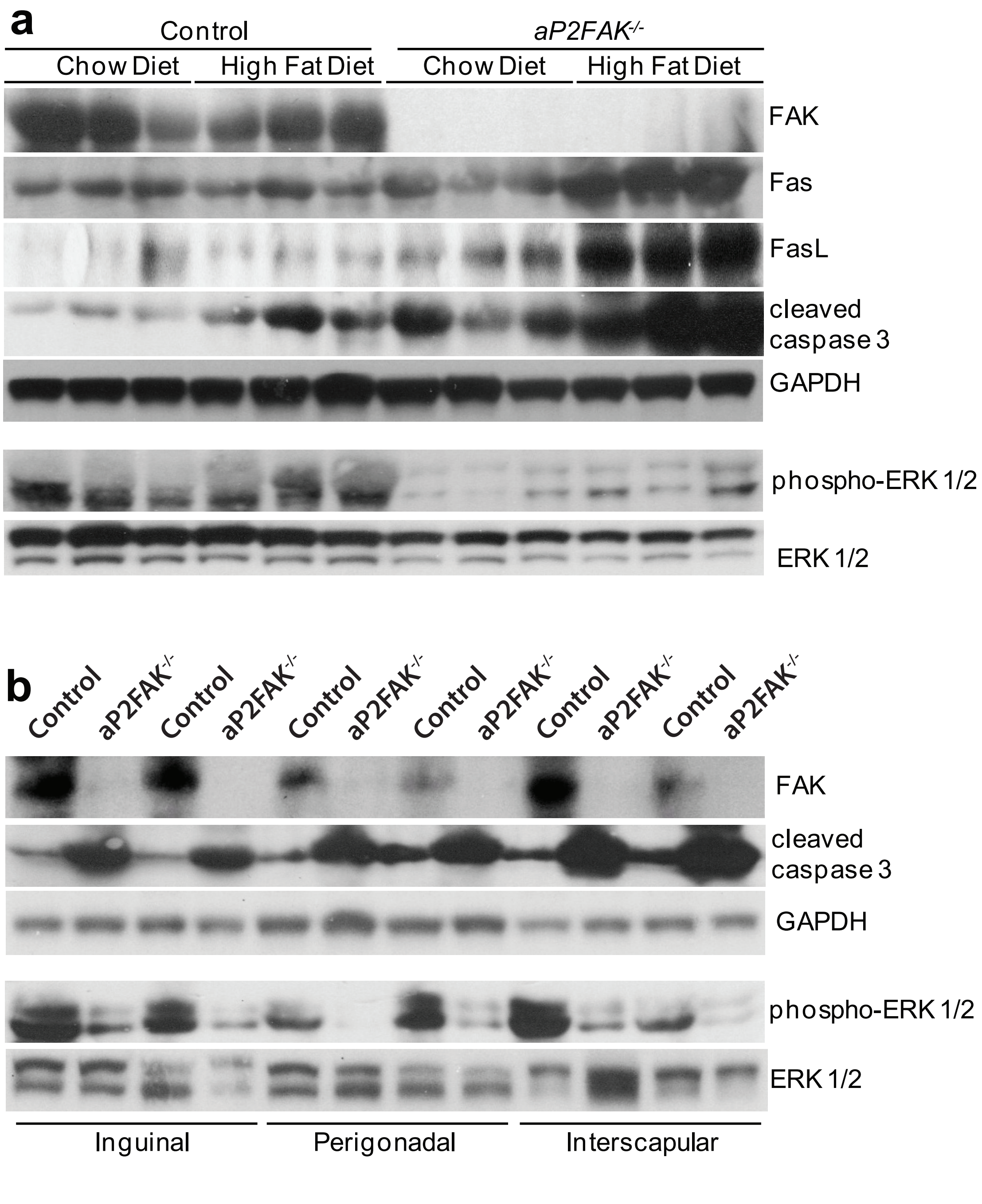

Supplementary Figure 4. FAK is required for adipocyte survival. (**a**) Increased pro-apoptotic and decreased cell survival signaling proteins in perigonadal fat of chow and HFD-fed *aP2FAK*$^{-/-}$ or littermate control mice. (**b**) Increased pro-apoptotic and decreased cell survival signaling proteins in inguinal, perigonadal and interscapular brown adipose tissue of *aP2FAK*$^{-/-}$ or littermate control mice. (**c**) Quantification of Ki67 staining in perigonadal fat from 20 week old mice (n = 4 control or 3 *aP2FAK*$^{-/-}$ mice). Data are mean ± SEM.

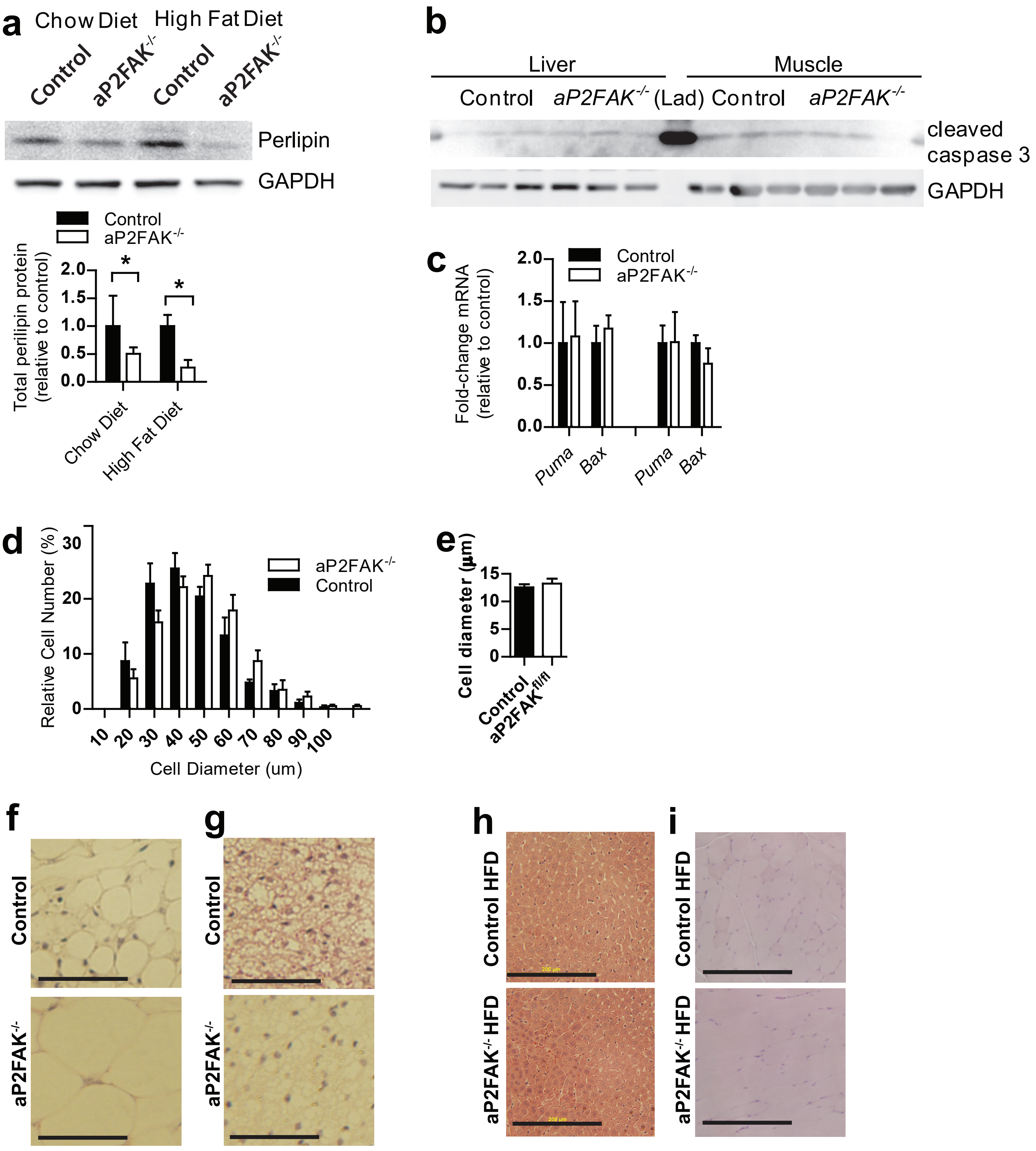

Supplementary Figure 5. FAK is required for adipocyte survival. (**a**) Western blot of perilipin protein in perigonadal fat of 20-24 week old chow and HFD-fed mice. (**b**,**c**) Western blot for cleaved caspase 3 in liver and muscle (Lad, protein ladder) (**b**), and relative gene expression of apoptosis mediators in liver and muscle (n = 4 mice) (**c**) of 20-24 week old mice. (**d**,**e**) Adipocyte cell size distribution (**d**) and diameter (**e**) from perigonadal fat pads of 4-6 week old mice (n = 3 mice). (**f**,**g**) Inguinal WAT (**f**) and interscapular BAT (**g**) sections stained with H&E (scale bar, 200 µm) from 20-24 week old mice. (**h**,**i**) Liver (**h**) and quadriceps muscle (**i**) sections stained with H&E from 20-24 week old HFD-fed mice (scale bar, 200 µm). Data are mean ± SEM. * $p < 0.05$ by Student's t-test.

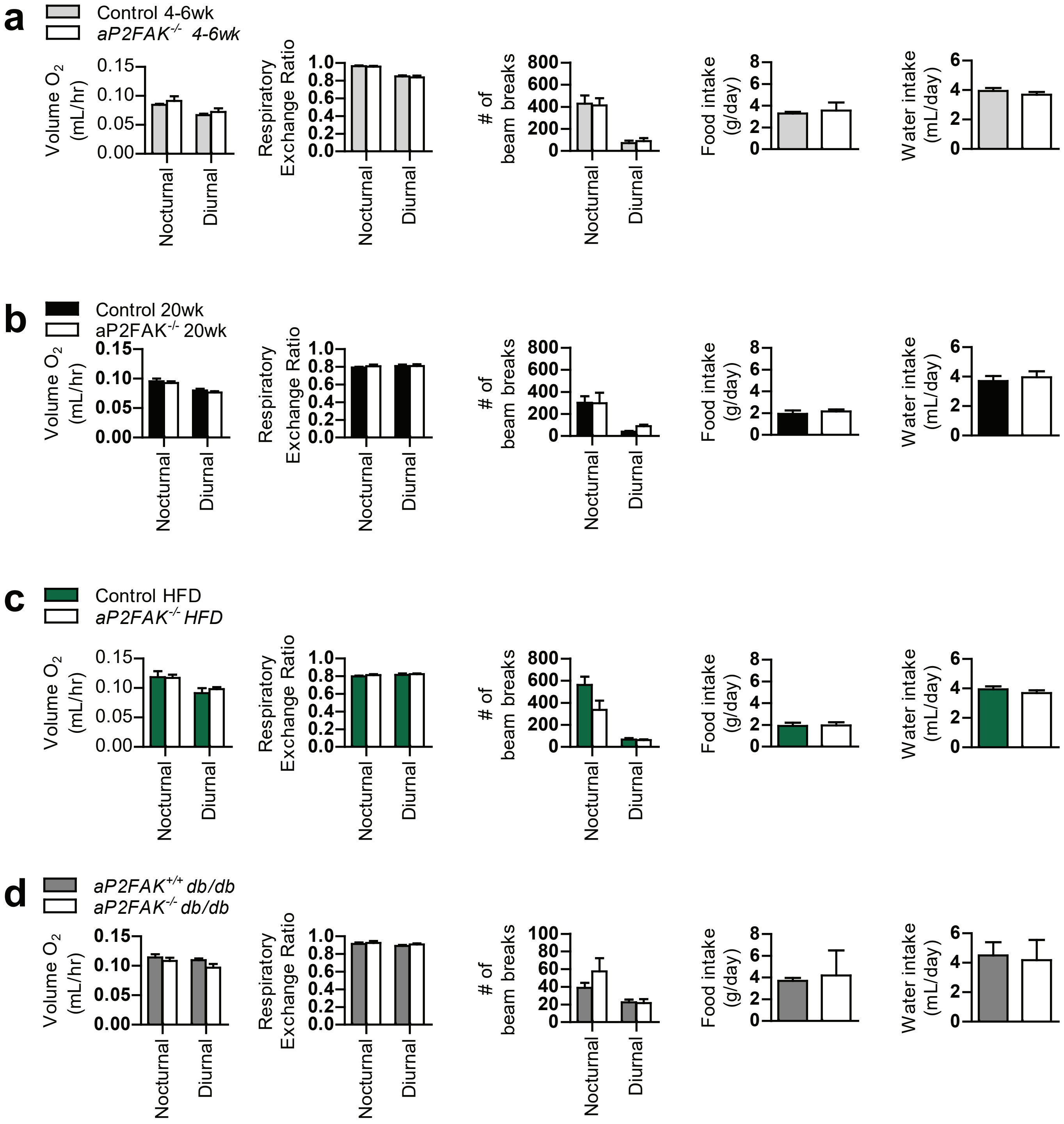

Supplementary Figure 6. Energy homeostasis in *aP2FAK$^{-/-}$* mice. (**a-d**) Energy expenditure measured by oxygen consumption, fuel utilization measured by respiratory expenditure ratio, ambulatory activity, food intake and water intake in 4-6 week old chow diet-fed mice (n = 4) (**a**), 20 week old chow diet-fed mice (n = 6) (**b**), HFD-fed mice (n = 4) (**c**), and mice on a *db/db* genetic background (n = 6) (**d**). Data are mean ± SEM.

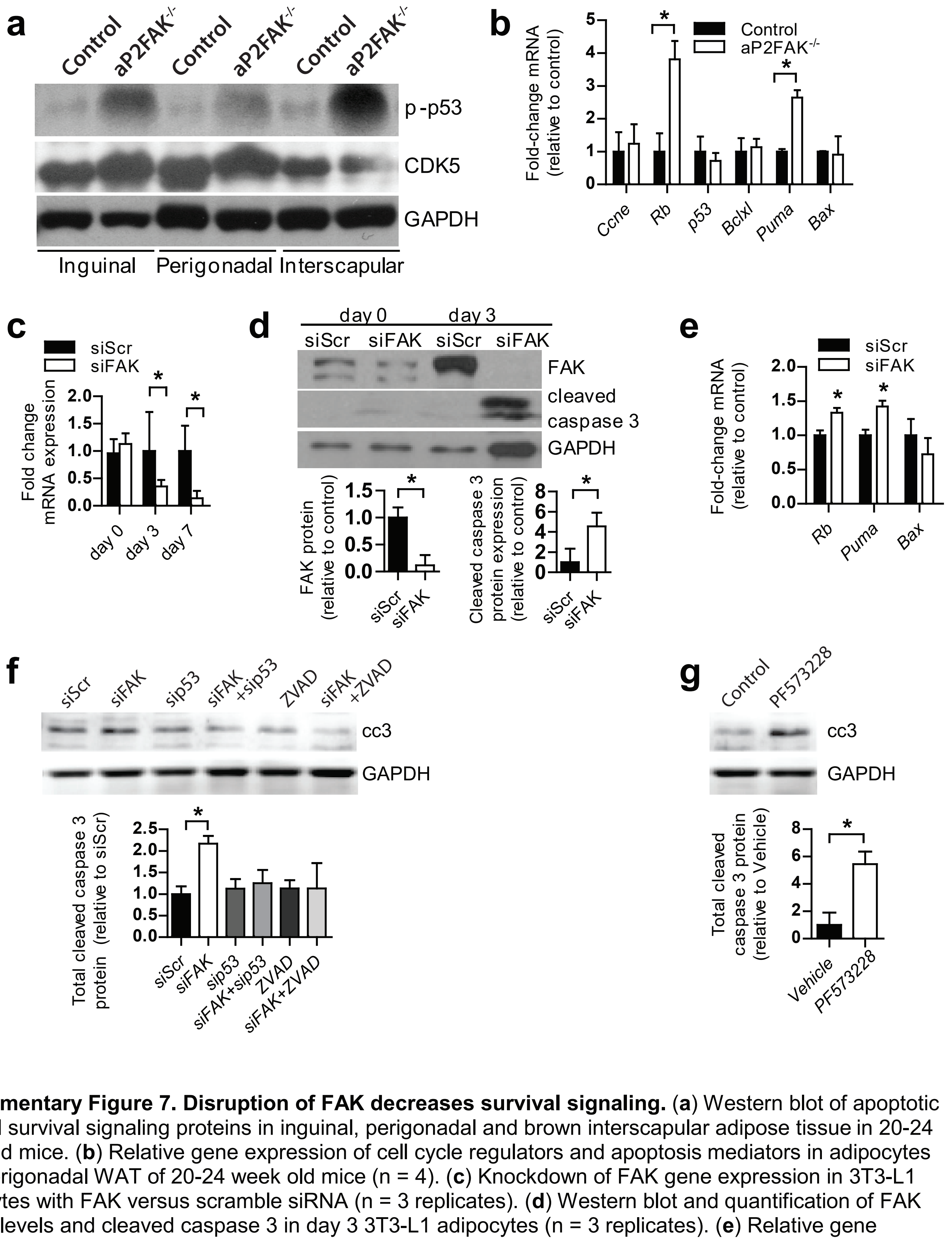

Supplementary Figure 7. Disruption of FAK decreases survival signaling. (**a**) Western blot of apoptotic and cell survival signaling proteins in inguinal, perigonadal and brown interscapular adipose tissue in 20-24 week old mice. (**b**) Relative gene expression of cell cycle regulators and apoptosis mediators in adipocytes from perigonadal WAT of 20-24 week old mice (n = 4). (**c**) Knockdown of FAK gene expression in 3T3-L1 adipocytes with FAK versus scramble siRNA (n = 3 replicates). (**d**) Western blot and quantification of FAK protein levels and cleaved caspase 3 in day 3 3T3-L1 adipocytes (n = 3 replicates). (**e**) Relative gene expression of cell cycle regulators and apoptosis mediators in 3T3-L1 adipocytes with FAK versus scramble siRNA (n = 3 replicates). (**f**) Western blot of cleaved caspase 3 in day 7 3T3-L1 adipocytes following treatment with scramble siRNA (siScr), FAK siRNA (siFAK), p53 siRNA (sip53) and/or ZVAD (n = 3). (**g**) Western blot of cleaved caspase 3 in 3T3-L1 adipocytes treated with FAK inhibitor PF573228 (n = 3 replicates). Data are mean ± SEM. * $p < 0.05$ by Student's t-test.

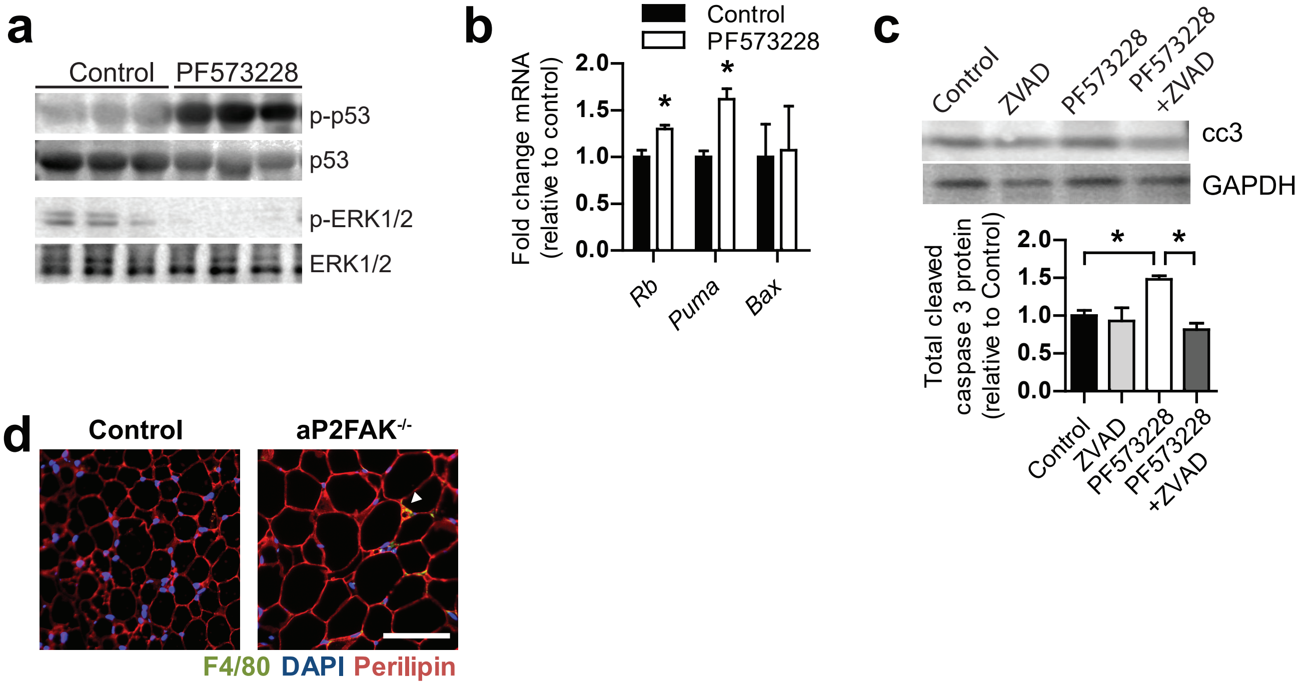

Supplementary Figure 8. Disruption of FAK decreases survival signaling. (**a**) Western blot for apoptotic and cell survival signaling proteins in primary mouse adipocytes treated with FAK inhibitor PF573228 or vehicle (Control) (n = 3 replicates). (**b**) Relative gene expression of cell cycle regulators and apoptosis mediators in primary adipocytes treated with FAK inhibitor PF573228 (n = 3 replicates). (**c**) Western blot of cleaved caspase 3 in primary adipocytes following treatment with FAK inhibitor PF573228 and/or ZVAD (n = 3 replicates). (**d**) Immunofluorescence for F4/80 (scale bar, 100 μm; arrows indicate positive nuclei) in perigonadal WAT sections from 20-24 week old mice. Data are mean ± SEM. * $p < 0.05$ by Student's t-test.

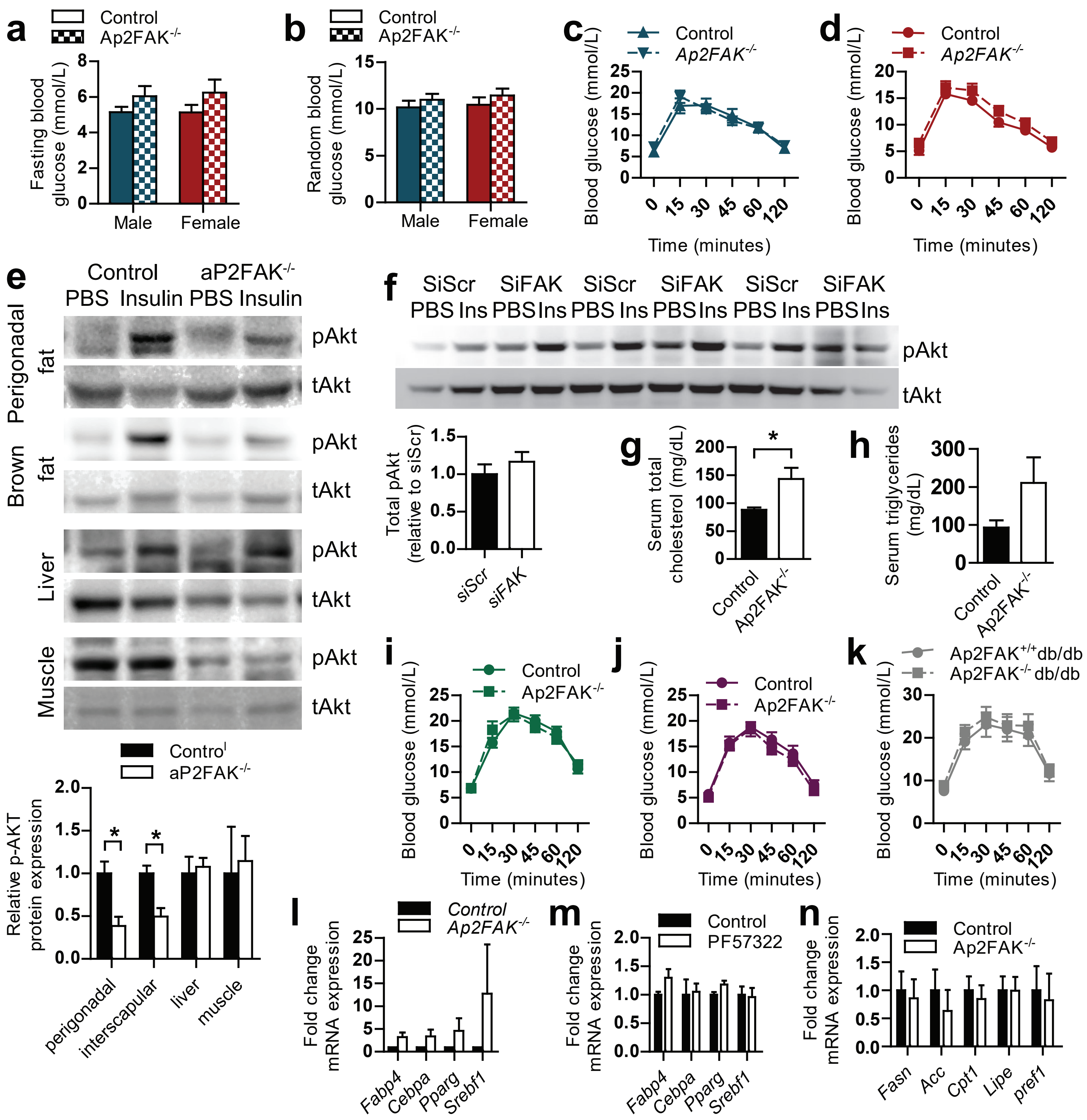

Supplementary Figure 9. Adipose tissue FAK is required for maintaining insulin sensitivity. (**a**,**b**) Fasting (**a**) and random (**b**) blood glucose in male (blue) and female (red) control or *aP2FAK*$^{-/-}$ mice at 4-6 weeks of age (n = 3). (**c**-**d**) I.p. glucose tolerance testing in male (**c**) and female (**d**) mice at 20-24 weeks of age (n = 10). (**e**,**f**) Representative Western blot with quantification of phosphorylated Akt (pAkt) protein levels, normalized to total Akt (tAkt) and relative to control mice in WAT, BAT, liver and muscle (n = 3 mice) (**e**) or differentiated 3T3-L1 adipocytes in scramble (siScr) vs FAK (siFAK) siRNA treated cells following insulin stimulation (n = 3 replicates) (**f**). (**g**,**h**) Fasting serum total cholesterol (**g**) and triglycerides (**h**) in 20-24 week old mice fed chow diet (n = 6). (**i**-**k**) I.p. glucose tolerance testing in 20-24 week old male (**i**) and female (**j**) mice fed HFD for 12-16 weeks (n = 9), and 6-8 week old mice on *db/db* genetic background (n = 6) (**k**). (**l**,**m**) Relative expression of adipogenic genes in inguinal WAT of 20-24 week old chow diet fed mice (n = 4 mice) (**l**) and primary mouse adipocytes treated with FAK inhibitor PF573228 (n = 3 replicates) (**m**). (**n**) Relative expression of lipolysis and lipogenesis genes in perigonadal WAT of 20-24 week old mice (n = 4). Data are mean ± SEM. * $p < 0.05$ by Student's t-test.

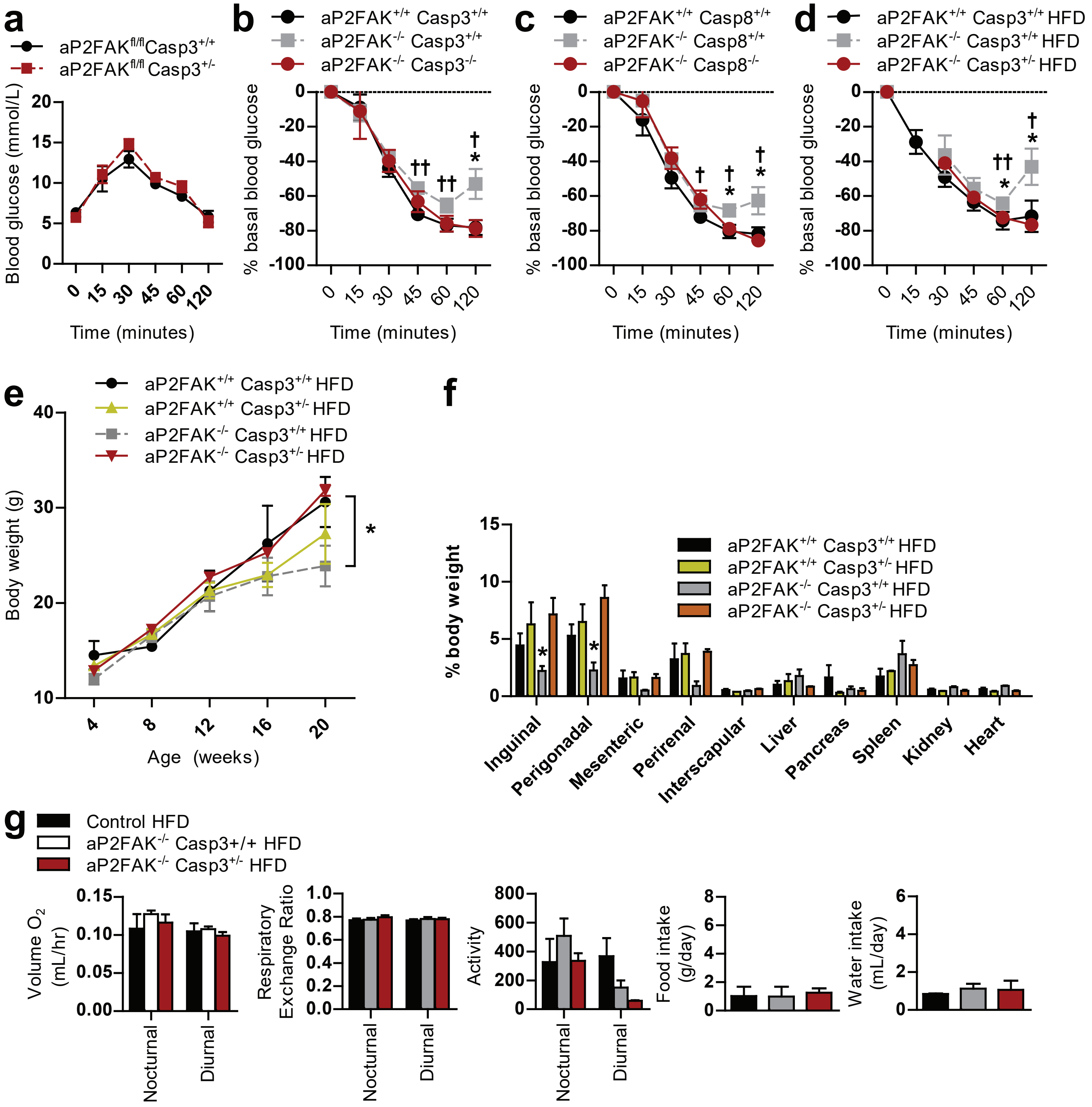

Supplementary Figure 10. Impairing apoptosis restores adipose tissue expansion and insulin sensitivity. (**a**) Glucose tolerance testing in *aP2FAK$^{-/-}$ Casp3$^{+/+}$* and *aP2FAK$^{-/-}$ Casp3$^{+/-}$* mice (n = 5). (**b-d**) Insulin tolerance testing in *aP2FAK$^{+/+}$ Casp3$^{+/+}$*, *aP2FAK$^{-/-}$ Casp3$^{+/+}$* and *aP2FAK$^{-/-}$ Casp3$^{-/-}$* mice (n = 3) (**b**), *aP2FAK$^{+/+}$ Casp8$^{+/+}$*, *aP2FAK$^{-/-}$ Casp8$^{+/+}$* and *aP2FAK$^{-/-}$ Casp8$^{-/-}$* mice (n = 6) (**c**) and *aP2FAK$^{-/-}$ Casp3$^{+/+}$*, *aP2FAK$^{-/-}$ Casp3$^{+/+}$* and *aP2FAK$^{-/-}$ Casp3$^{+/-}$* mice on HFD (n = 5) (**d**). * $p < 0.05$ *aP2FAK$^{-/-}$ Casp3$^{-/-}$*, *aP2FAK$^{-/-}$ Casp8$^{-/-}$, or aP2FAK$^{-/-}$ Casp3$^{+/-}$* versus *aP2FAK$^{-/-}$ Casp3$^{+/+}$* or *aP2FAK$^{-/-}$ Casp8$^{+/+}$* mice. † $p < 0.05$, †† $p < 0.01$ *aP2FAK$^{-/-}$ Casp3$^{+/+}$* or *aP2FAK$^{-/-}$ Casp8$^{+/+}$* versus *aP2FAK$^{+/+}$ Casp3$^{+/+}$* or *aP2FAK$^{+/+}$ Casp8$^{+/+}$* mice. (**e-f**) Body weight (**e**) and composition (**f**) in littermate control or *aP2FAK$^{-/-}$ Casp3$^{+/-}$* mice fed HFD (n = 3). (**g**) Energy expenditure measured by oxygen consumption, fuel utilization measured by respiratory expenditure ratio, ambulatory activity, food intake and water intake in *aP2FAK$^{+/+}$ Casp3$^{+/+}$*, *aP2FAK$^{-/-}$ Casp3$^{+/+}$* and *aP2FAK$^{-/-}$ Casp3$^{+/-}$* mice (n = 4). Experiments were performed at 20-24 weeks of age. Data are mean ± SEM. * $p < 0.05$ by Student's t-test.

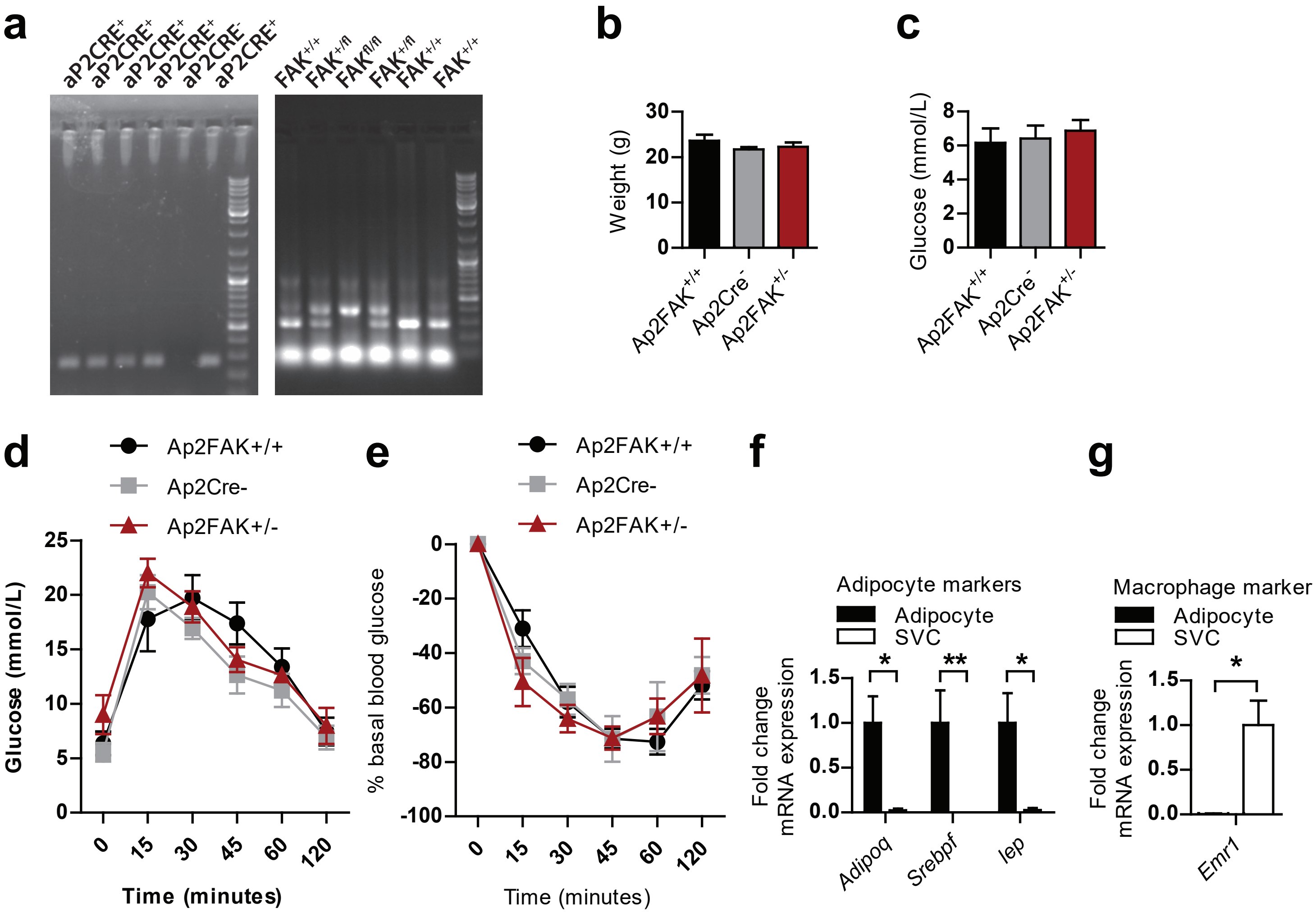

Supplementary Figure 11. Mouse genotyping and adipocyte isolation. (**a**) PCR genotyping results for aP2CRE and FAK$^{fl/fl}$ mice. (**b-e**) Body weight (n = 6) (**b**), fasting blood glucose (n = 6) (**c**), glucose tolerance testing (n = 4) (**d**) and insulin tolerance testing (n = 3) (**e**) in 20 week old *aP2FAK*$^{+/+}$, *aP2Cre*$^{-}$ and *aP2FAK*$^{+/-}$ mice. (**f,g**) Relative expression of adipocyte-specific Adiponectin gene (*Adipoq*) or genes preferentially expressed in adipocytes (*Srebpf*, *Lep*) in stromal vascular cells (SVC) versus isolated mature adipocytes (**f**); and relative expression of macrophage F4/80 (*Emr1*) in SVC versus adipocyte fraction (n = 5 mice) (**g**). Data are mean ± SEM. * $p < 0.05$, ** $p < 0.01$ by Student's t-test.

Supplementary Figure 12. Uncropped scans of Western blots.
Western blots for Figure 1a.

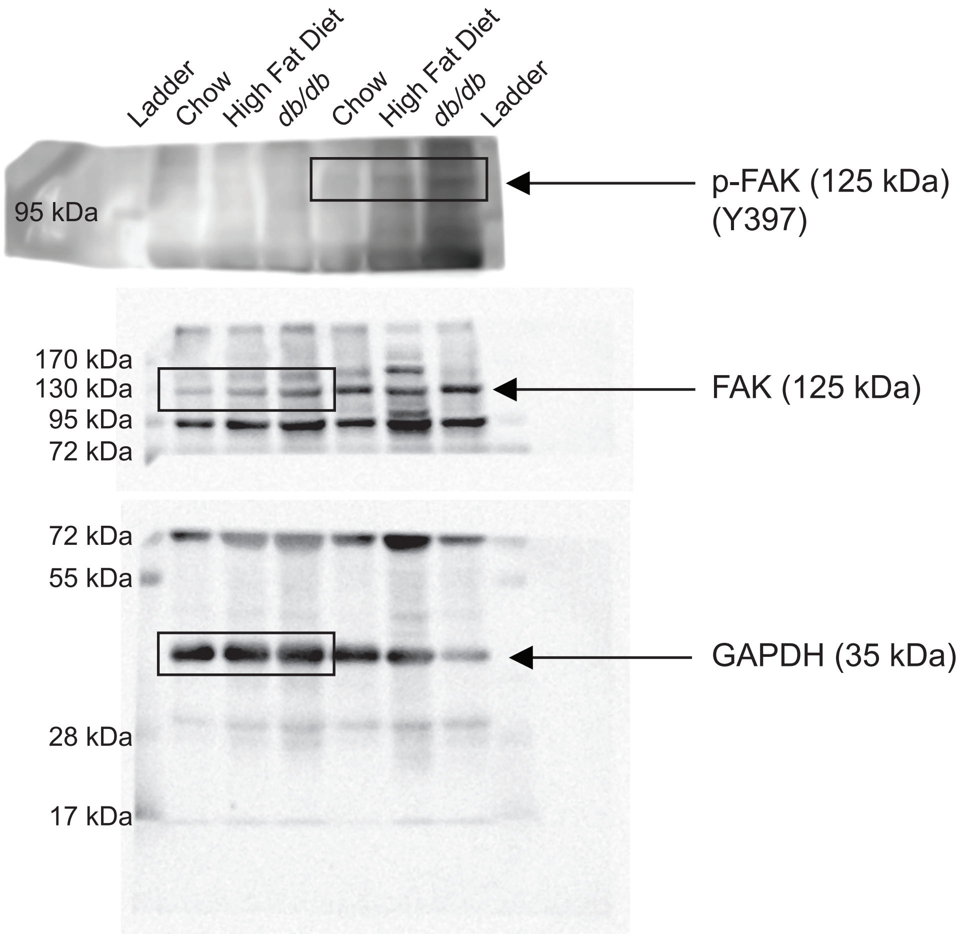

Western blots for Figure 1b.

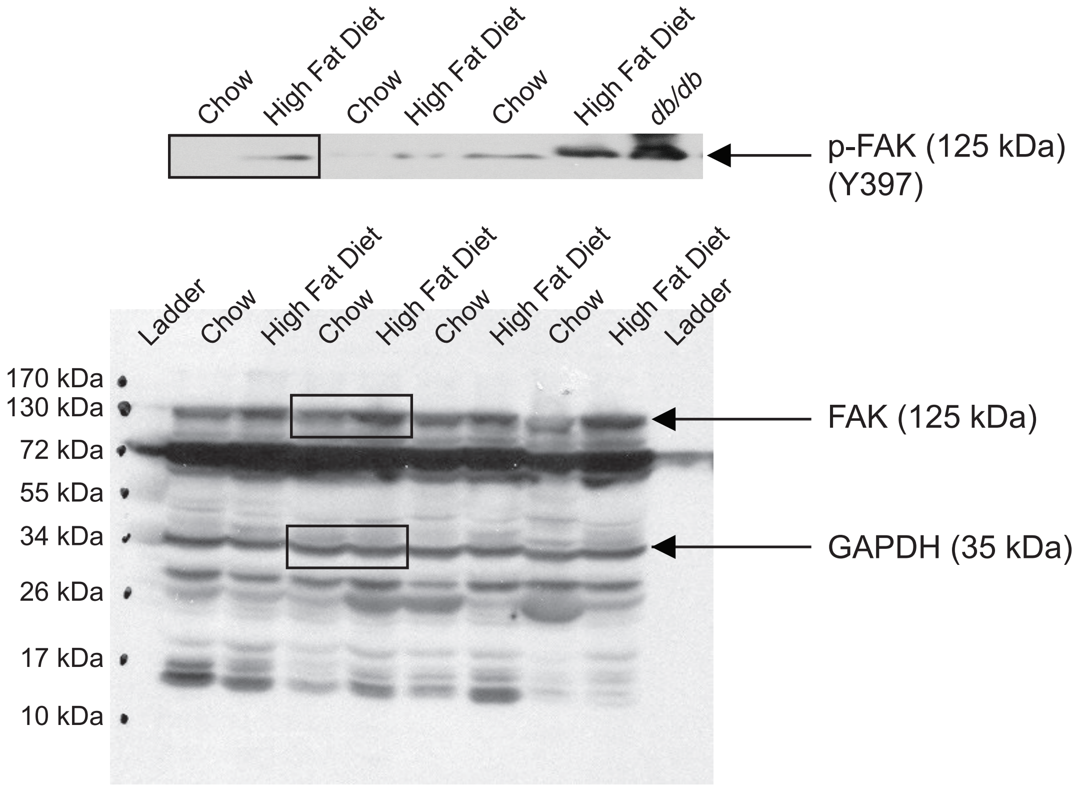

Western blots for Figure 1c.

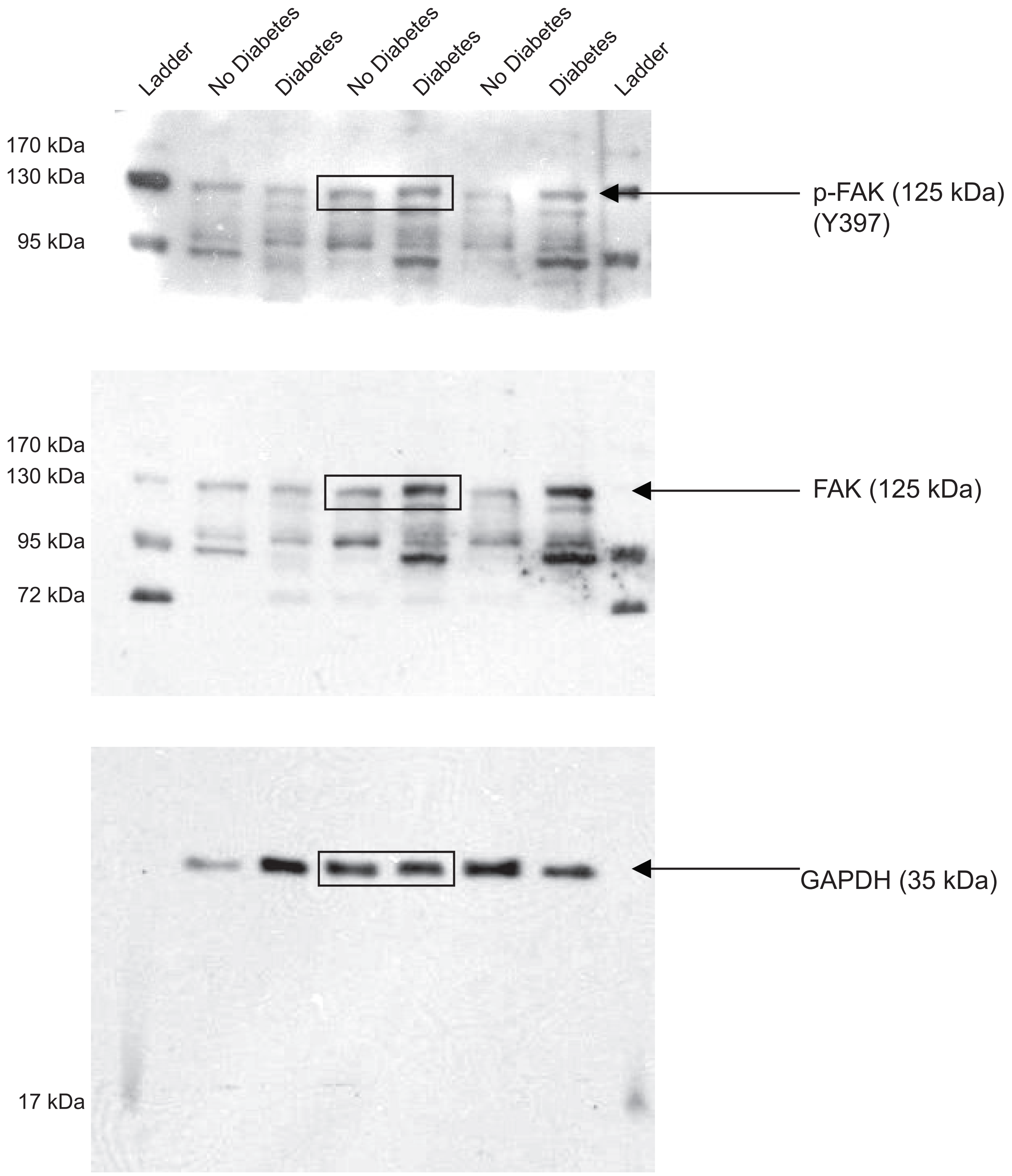

Western blots for Figure 4c.

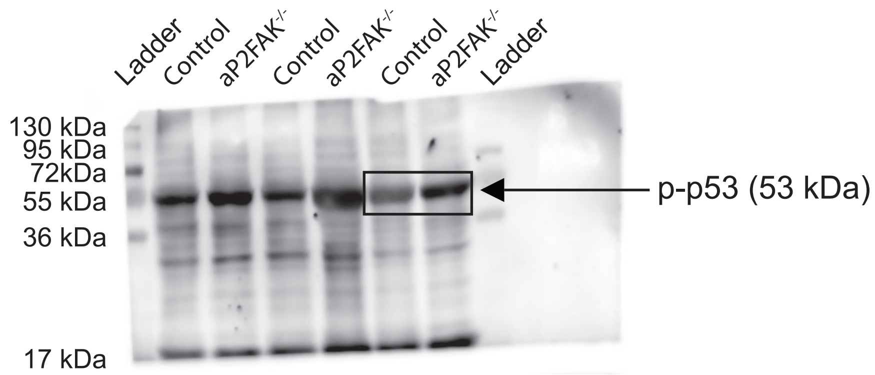

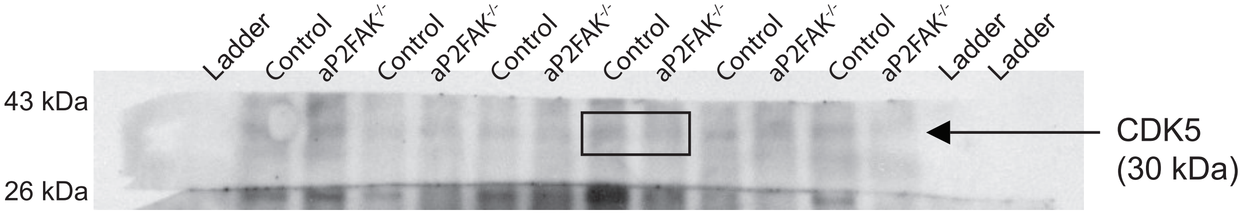

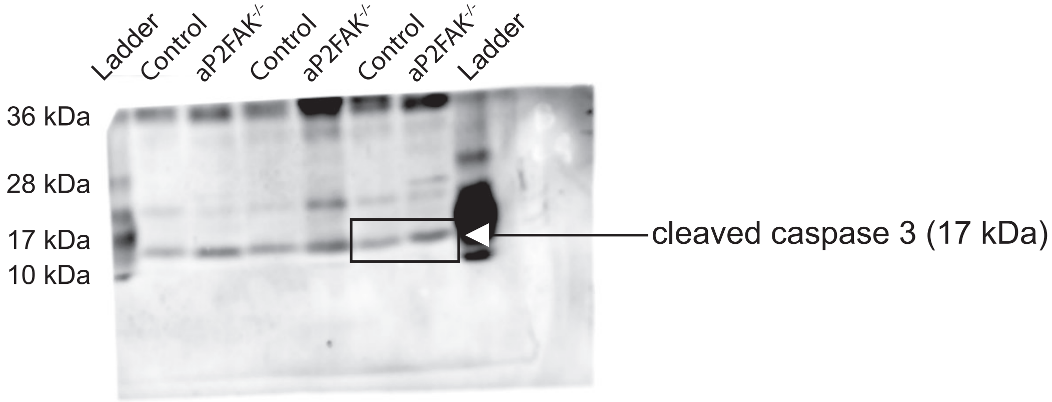

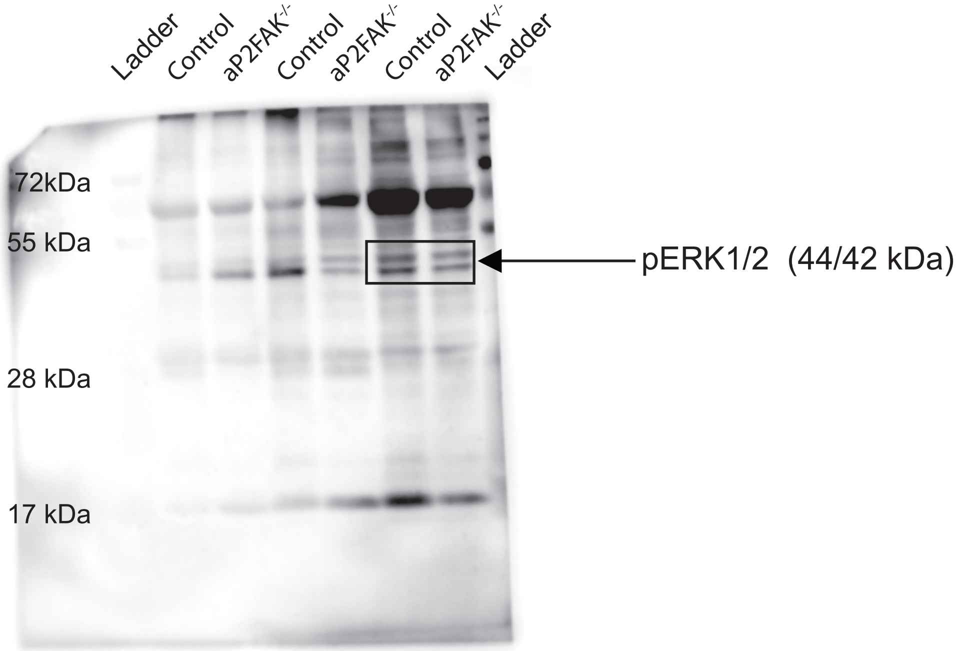

Western blots for Figure 4c.

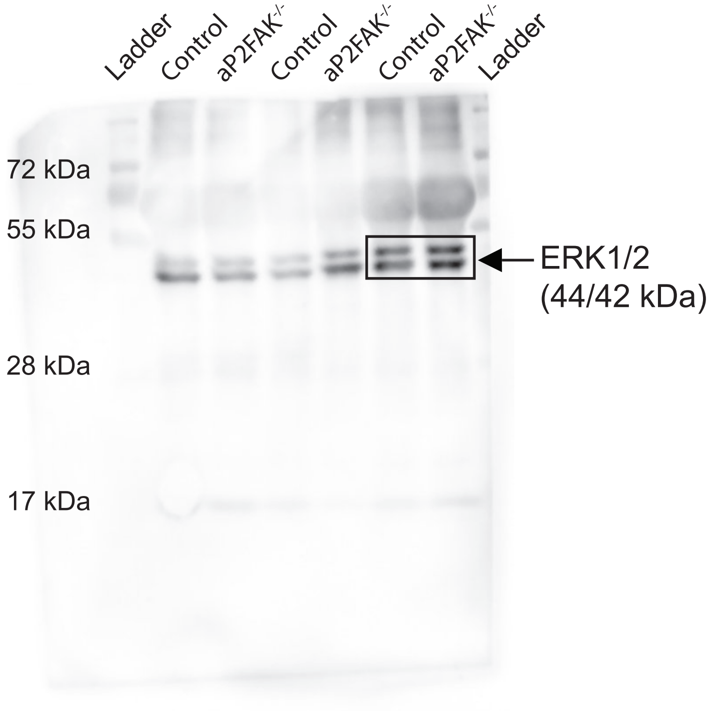

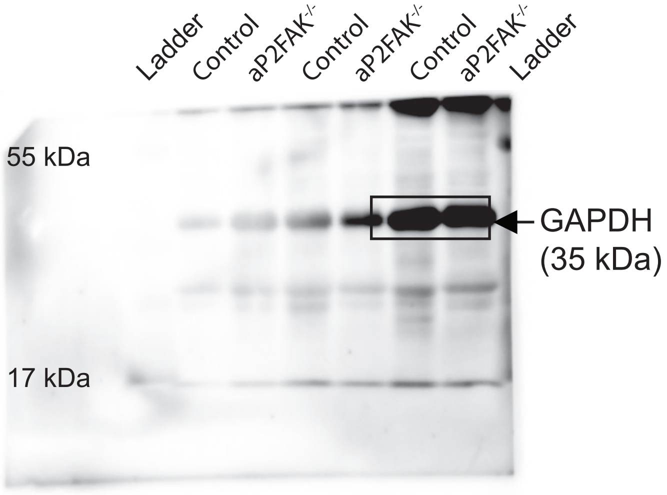

Supplementary Table 1. Sequence of primers used for quantitative RT-PCR

Primer	Sequence
18s forward	5'-AGTCCCTGCCCTTTGTACACA-3'
18s reverse	5'-CGATCCGAGGGCCTCACTA-3'
Acc forward	5’-CTCCAGGACAGCACAGATCA-3’
Acc reverse	5’-TGACTGCCGAAACATCTCTG-3’
Adipoq forward	5'-GGAACTTGTGCAGGTTGGAT-3'
Adipoq reverse	5'-GCTTCTCCAGGCTCTCCTTT-3'
Bax forward	5’-TGTTTGCTGATGGCAACTTC-3’
Bax reverse	5’-GATCAGCTCGGGCACTTTAG-3’
Bclxl forward	5’-GCTGGGACACTTTTGTGGAT-3’
Bclxl reverse	5’-TGTCTGGTCACTTCCGACTG-3’
Ccne forward	5'-CTGAGAGATGAGCACTTTCTG-3'
Ccne reverse	5'-GAGCTTATAGACTTCGCACACCT-3'
Cebpa forward	5'-AAGAACAGCAACGAGTACCGG-3'
Cebpa reverse	5'-CATTGTCACTGGTCAGCTCCA-3'
Cpt1 forward	5’-GCAGAGCACGGCAAAATGA-3’
Cpt1 reverse	5’-CTTTCGACCCGAGACCTT-3’
Emr1 forward	5'-CTTTGGCTATGGGCTTCCAGTC-3'
Emr1 reverse	5'-GCAAGGAGGACAGAGTTTATCGTG-3'
Fabp4 forward	5'-GACGACAGGAAGGTGAAGAG-3'
Fabp4 reverse	5'-ACATTCCACCACCAGCTTGT-3'
Fasn forward	5’-CTCTGATCAGTGGCCTCCTC-3’
Fasn reverse	5’-AGCTGCAGTTTGGTCTGAAC-3’
Il6 forward	5'-CTCTGGGAAATCGTGGAAATG-3'
Il6 reverse	5'-AAGTGCATCATCGTTGTTCATACA-3'
Lep forward	5'-TTCACACACGCAGTCGGTAT-3'
Lep reverse	5'-GCTGGTGAGGACCTGTTGAT-3'
Lpl forward	5’-GCCCAGCAACATTATCCAGT-3’
Lpl reverse	5’-GGTCAGACTTCCTGCTACGC-3’
Pparg forward	5'-GCCCTTTGGTGACTTTATGG-3'
Pparg reverse	5'-CAGCAGGTTGTCTTGGATGT-3'
Pref1 forward	5’-AACAATGGAACTTGCGTGGAC-3’
Pref1 reverse	5’-TGCGGCTACGATCTCACAGA-3’
Ptk2 forward	5'-AACTCAAAATCGCTGGTGCT-3'
Ptk2 reverse	5'-TGCCTTGCTTTTCACTGTTG-3'
Puma forward	5’-ATGCCTGCCTCACCTTCATCT-3’
Puma reverse	5’-AGCACAGGATTCACAGTCTGGA-3’
Rb forward	5'-TACACTCTGTGCACGCCTTC-3'
Rb reverse	5'-TTCACCTTGCAGATGCCATA-3'
Srebf1 forward	5'-GATCAAAGAGGAGCCAGTGC-3'
Srebf1 reverse	5'-TAGATGGTGGCTGCTGAGTG-3'

Tnf forward	5'-CTGTGAAGGGAATGGGTGTT-3'
Tnf reverse	5'-TTGGACCCTGAGCCATAATC-3'
